# Replicatively senescent human fibroblasts reveal a distinct intracellular metabolic profile with alterations in NAD+ and nicotinamide metabolism

**DOI:** 10.1038/srep38489

**Published:** 2016-12-07

**Authors:** Emma L. James, James A. E. Lane, Ryan D. Michalek, Edward D. Karoly, E. Kenneth Parkinson

**Affiliations:** 1 Centre for Clinical & Diagnostic Oral Sciences, Institute of Dentistry, Barts and the London School of Medicine and Dentistry, Queen Mary University of London, Turner Street, London, E1 2AD, UK; 2 Metabolon, Inc. 617 Davis Drive, Suite 400, Durham, NC, 27713, USA.

## Abstract

Cellular senescence occurs by proliferative exhaustion (PEsen) or following multiple cellular stresses but had not previously been subject to detailed metabolomic analysis. Therefore, we compared PEsen fibroblasts with proliferating and transiently growth arrested controls using a combination of different mass spectroscopy techniques. PEsen cells showed many specific alterations in both the NAD+ *de novo* and salvage pathways including striking accumulations of nicotinamide mononucleotide (NMN) and nicotinamide riboside (NR) in the amidated salvage pathway despite no increase in nicotinamide phosphoribosyl transferase or in the NR transport protein, CD73. Extracellular nicotinate was depleted and metabolites of the deamidated salvage pathway were reduced but intracellular NAD+ and nicotinamide were nevertheless maintained. However, sirtuin 1 was downregulated and so the accumulation of NMN and NR was best explained by reduced flux through the amidated arm of the NAD+ salvage pathway due to reduced sirtuin activity. PEsen cells also showed evidence of increased redox homeostasis and upregulated pathways used to generate energy and cellular membranes; these included nucleotide catabolism, membrane lipid breakdown and increased creatine metabolism. Thus PEsen cells upregulate several different pathways to sustain their survival which may serve as pharmacological targets for the elimination of senescent cells in age-related disease.

Senescent cells accumulate in a variety of pathologies^1^ and ageing and can modulate them^2^,^3^,^4^. Cellular senescence can occur by a variety of mechanisms including telomere attrition, following proliferative exhaustion (PEsen), otherwise known as replicative senescence. Many senescence mechanisms, including PEsen involve the production of DNA double strand breaks (DSBs), which may result from telomere uncapping^5^,^6^ or from the generation of oxidative DNA damage and stalled replication forks in S phase^1^ but this is not always the case^7^. The early events in the establishment of senescence are transiently reversible^8^,^9^ but the failure to repair DSBs (IrrDSBs) leads eventually to the permanent cell cycle arrest defined as senescence and to the production of an array of secreted proteins termed the senescence-associated secretory phenotype (SASP refs 7 and 10). The SASP includes a variety of cytokines some of which are thought to be involved in senescent cell clearance^3^,^4^,^11^ but if PEsen cells avoid immune surveillance they are capable of remaining viable for up to 3 years in the post-mitotic phase^12^ despite sustaining persistent DNA damage^13^. The mechanisms by which senescent cells remain viable are still very unclear, although they are resistant to a variety of apoptotic signals^1^ and drugs that target senescent cell survival mechanisms, termed senolytics, have recently been shown to selectively clear senescent cells and rejuvenate tissues^14^,^15^,^16^,^17^.

There is accumulating evidence demonstrating the regulation of senescence and the SASP by metabolic enzymes^7^,^18^,^19^,^20^,^21^ but these studies have largely concentrated in the induction of senescence by oncogenic stress, otherwise known as oncogene-induced senescence (OIS) and/or cell types that senesce by mechanisms other than PE. Therefore, an unbiased metabolic profile of PEsen cells has not yet been established. There are various approaches that can be used to analyse the metabolomes of cells and body fluids and the strengths and weaknesses of these different techniques have recently been reviewed^22^. We have used a variety of mass spectroscopy techniques coupled with a library of over 3000 standards^23^ to identify the intracellular metabolites of human fibroblasts and for the first time, produce an unbiased assessment of the metabolic state of these biologically important cells.

We have established that PEsen fibroblasts modify their extracellular metabolites in a manner that overlaps considerably with that of the same cells induced to senesce by irreparable DNA damage and the metabolic profile of ageing humans *in vivo*^23^. We also reported previously that the intracellular metabolic profile surprisingly showed a shift of energy metabolism away from the tri-carboxylic acid (TCA) cycle and towards glycolysis and the pentose phosphate pathway^23^ and more recent evidence suggests that the shift to glycolysis may dependent on mitochondrial dysfunction^24^. In addition, we reported evidence of a strong redox homeostasis response that is consistent with relatively low levels of cells positive for nuclear 8-hydroxy-2-deoxyguanosine compared to DNA damage foci in established PEsen cells^23^,^25^.

We now report that PEsen fibroblasts display further intracellular specific metabolic changes, independently of growth arrest, which although complex and numerous, would conventionally be expected to generate alternative energy sources or otherwise protect the cells against oxidative damage and support cell survival. In particular, PEsen cells maintained NAD+ and nicotinamide levels despite showing no increase in the rate-limiting enzymes for their production and greatly suppressed sirtuin levels. However, there was evidence of increased nicotinamide turnover suggesting that compensatory mechanisms are activated in senescent cells. Such mechanisms may offer targets for the development of senolytic drugs in the future.

## Results

### Characteristion of PEsen cells and controls

The NHOF-1 oral fibroblast line has been characterised previously^23^,^25^ and examples of this characterisation and quantitation of the fraction of cells expressing senescence-associated beta galactosidase (SA-βGal) are shown in Supplementary Fig. S1. In addition to the quantitation of the Ki67 and large 53BP1 foci reported previously^23^ the data shows that a high fraction of the PEsen cells but not the growing, quiescent or confluent cells express SA-βGal (Supplementary Fig. S1A) and as reported previously^23^ high numbers of cells expressing large 53BP1 foci indicative of unrepaired DNA double strand breaks (Supplementary Fig. S1B). The PEsen cells but not the growing, quiescent or confluent cells also showed extremely low levels of SIRT1 (see below) as reported by other groups as markers of senescence^26^. However, the quiescent and confluent transiently growth-arrested controls displayed nearly equivalent fractions of Ki67-positive cells to PEsen cells (Supplementary Fig. S1B), showing that all proliferation arrested groups possessed equivalent numbers of cells that were out of cycle (see also ref. 23).

### Principal component and cluster analysis

**Figure 1:**
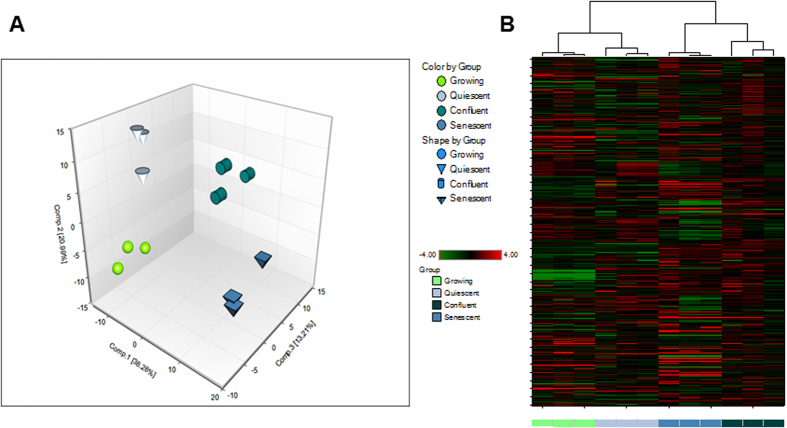
Principal component and cluster analysis of PEsen versus controls. The Figure shows (**A**) Principal component analysis of growing, quiescent, confluent and PEsen (senescent) cells showing that all four groups have distinct metabolic profiles. (**B**) Dendrograms representing cluster analysis of the same experimental groups showing that confluent cells cluster closer to senescent cells than growing or quiescent cells.

Principal component analysis (PCA) is a mathematical procedure that uses an orthogonal transformation to convert a set of observations of possibly correlated variables into a set of values of linearly uncorrelated variables called principle components. PCA revealed a distinct separation between conditions in cell pellet samples (Fig. 1A) that may be indicative of unique biochemical signatures associated with growing, confluent, quiescent, and senescent cells. Interestingly, confluent and PEsen cell pellet and media samples sorted together by hierarchical clustering (Fig. 1B) suggesting their metabolic profile may be more similar to one another than growing and quiescent counterparts. The results indicated that confluent cultures may be a much tighter control for metabolites specifically associated with PEsen than either growing or quiescent cultures. However, for the purpose of this study we only considered metabolites that differed significantly between PEsen cultures and all three control groups, as these were the most likely to be independent of cell cycle arrest (growing), nutrient and growth factor depletion, slowed metabolic rate (quiescent/serum-starved) and changes in cell shape (confluent). In all four groups 380 metabolites were detected and only 13% were specifically altered in PEsen cells. Of these 37 accumulated and 13 were depleted and are described below. The metabolites listed are those that differ with statistical significance between PEsen cells and all 3 control groups, as determined by ANOVA with Tukey’s post hoc analysis. The false discovery rates (FDRs) for most of these comparisons was <0.1.

### Redox homeostasis

**Figure 2:**
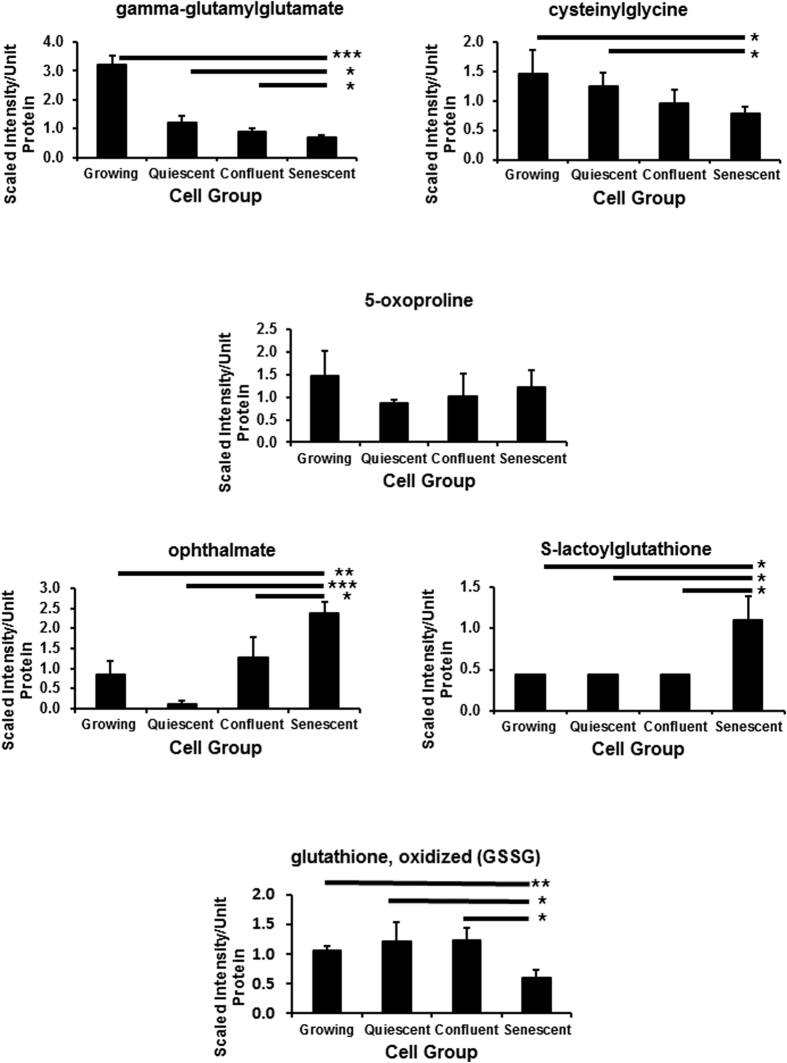
Modulation of intracellular redox metabolites in PEsen NHOF-1 cells relative to growing, quiescent and confluent cells. The Figure shows the gamma-glutamyl redox homeostasis pathway and levels of each metabolite normalised to cell protein content +/− standard deviation in growing, quiescent, confluent and PEsen NHOF-1 oral fibroblasts. N = 3 per cell group. *p, 0.1 > 0.05, *p < 0.05, **p < 0.01, ***p < 0.001 with a 1 way ANOVA and Tukey’s post hoc analysis.

The redox homeostasis metabolites specifically altered in PEsen cells are summarised in (Supplementary Table S1a) and the FDRs (q values) ranged from <0.06 to <0.27 for the different comparisons (Supplementary Table S1b). Gamma glutamyl amino acids (GGAAs) play an important role in regulating the exchange of intra-and extracellular glutathione as well as amino acid uptake. Gamma-glutamylphenylalanine and gamma-glutamylalanine were elevated in PEsen cells relative to the three control groups^23^ but multiple GGAAs including gamma-glutamylglutamate (Fig. 2) and gamma-glutamylglutamine^23^ and cytsteinylglycine (Fig. 2) were diminished in all three conditions compared to growing cells but this was more marked in PEsen cells (Fig. 2). However, 5-oxoproline levels that would be indicative of decreased GGAA import and catabolism did not differ between PEsen cells and the controls (Fig. 2). PEsen cells exhibited a strong trend for reduced levels of cysteine compared to growing, confluent, and quiescent counterparts^23^. This finding may reflect decreased synthesis considering cystathionine (Supplementary Fig. S2) also showed a slight trend for reduction in these cells, or may arise from decreased uptake as suggested by high cysteine levels in the conditioned media^23^. PEsen cells exhibited significantly higher levels of the tripeptide ophthalmate (Fig. 2, Supplementary Table S1) which is an analogue of glutathione in which cysteine has been replaced by 2-aminobutyrate. Cysteine-glutathione disulfide (an internal marker of reactive oxygen species), and cysteine sulfinic acid (generated from the irreversible oxidation of cysteine), both were elevated in PEsen cells and as reported previously in their conditioned media^23^. Oxidized glutathione (GSSG) (Fig. 2) and to a lesser extent reduced glutathione^23^ were diminished in PEsen cells (Fig. 2) and PEsen cells possessed detectable levels of S-lactoylglutathione (Fig. 2, Supplementary Table S1). The lipid peroxidation products 13-HODE^+^9-HODE and 4-hydroxy-nonenal-glutathione (a surrogate for 4-hydroxynonenal) accumulated in PEsen cells, although they also accumulated in confluent cells as well (Supplementary Fig. S2). Finally, there was also a trend towards low levels of alpha-tocopherol in PEsen cells compared to the controls (Supplementary Fig. S2) but this did not reach statistical significance.

Several other amino acids related to redox homeostasis were specifically elevated in PEsen cells including methionine sulphoxide (Supplementary Fig. S3, Supplementary Table S1). S-adenosylmethione (SAM) and its metabolite S-adenosylhomocysteine (SAH) as well as pipecolate were also specifically depleted in PEsen cells compared to growing controls and either quiescent or controls respectively (Supplementary Fig. S3, Supplementary Table S1).

NADPH was also elevated in PEsen cells but unlike the other redox homeostasis metabolites it was elevated in all conditions of growth arrest (Supplementary Fig. S3).

### Tryptophan and NAD+ metabolism

Tryptophan is metabolised by enzymes such as tryptophan 2, 3, dioxygenase to produce kynurenine and this in turn is further metabolised to quinolinic acid which can act a substrate for the enzyme quinolate phosphoribosyltransferase to produce NAD^+^ by the *de novo* pathway and as we reported previously kynurenine is strikingly and specifically elevated in PEsen cells^23^.

**Figure 3:**
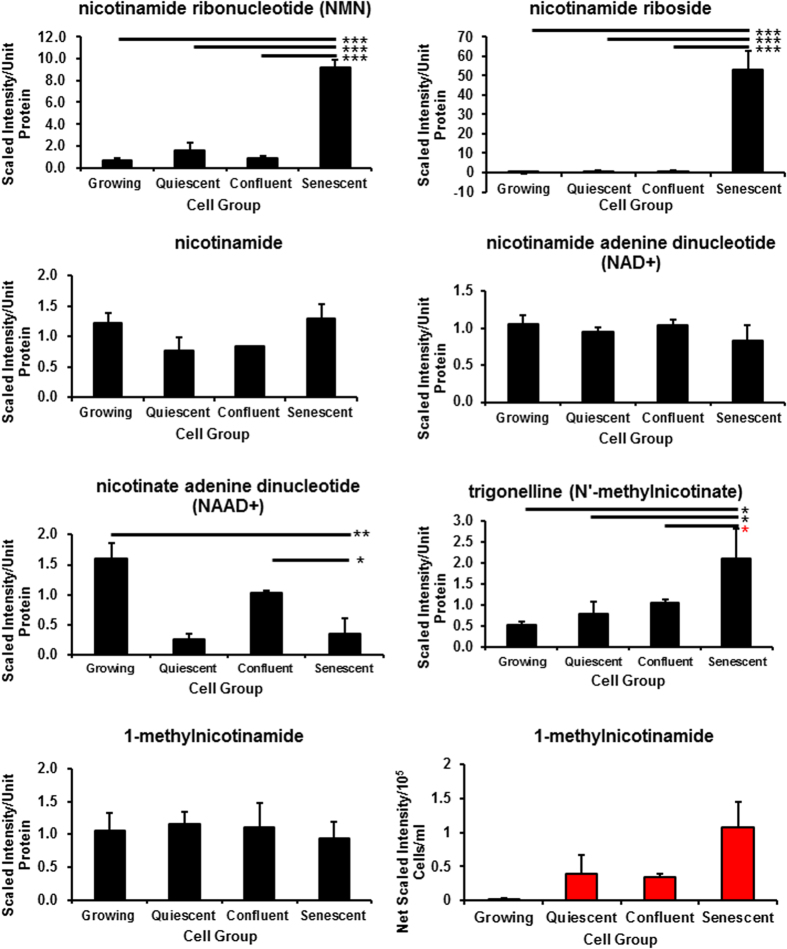
NAD+ and nicotinamide levels are sustained in PEsen cells: Evidence for alterations of the NAD+ salvage pathways. The Figure shows the alteration of intracellular (blue bars) and extracellular (red bars) metabolites within in PEsen cells and their controls in the NAD+ salvage pathways. Nicotinate adenine dinucleotide (NAAD+) was specifically depleted in PEsen cells whilst NAD+ levels and nicotinamide levels within the cell are sustained, as was the metabolic product of nicotinamide N-methyltransferase, 1-methyl nicotinamide and extracellular 1-methyl nicotinamide was elevated in PEsen. On the other hand the metabolic products of nicotinamide phosphoribosyl transferase, nicotinamide riboside and nicotinamide ribonucleotide (NMN), are strikingly and specifically elevated in PEsen cells and the breakdown product of nicotinate, trigonelline (N′-methylnicotinate) was also elevated. Levels of each metabolite normalised to cell protein content +/− standard deviation in growing, quiescent, confluent and PEsen NHOF-1 oral fibroblasts. N = 3 per cell group. The symbols are the same as for Fig. 2.

**Figure 4:**
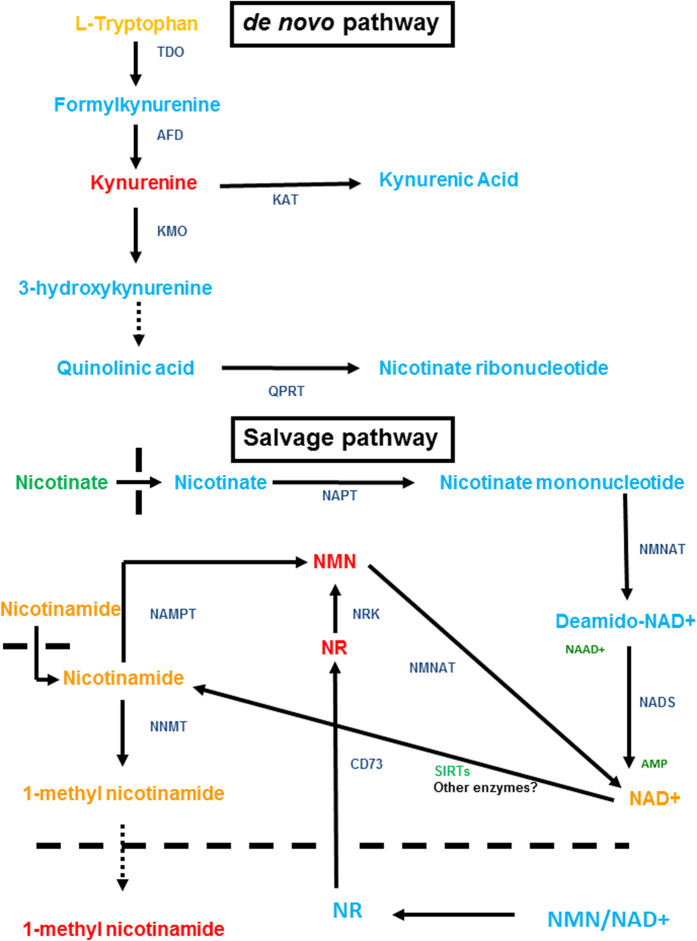
NAD+ *de novo* and salvage pathways. The figure is adapted from ref. 31 and summarises the key features of both the *de novo* pathway whereby tryptophan is metabolised to kynurenine and in the presence of the appropriate enzymes to NAD+ and the salvage pathway whereby NAD+ is maintained by the metabolism of the two vitamin B3 forms (nicotinate and nicotinamide). Detected elevated metabolites are shown in red, depleted metabolites in green, unchanged metabolites in orange and undetectable/undetected metabolites in light blue. Key enzymes are shown in blue. Abbreviations: TDO, Tryptophan 2,3-dioxygenase; AFD, Arylformamidase; KAT, Kynurenine amino transferase; KMO, Kynurenine 3-monooxygenase; QPRT, Quinolate phosphoribosyltransferase; NAPT, Nicotinate phosphoribosyltransferase; NNAT, nicotinate-nucleotide adenylyltransferase; NADS, NAD synthetase; NMNAT, NMN adenylyltransferase; NRK, Nicotinamide riboside kinase; NAMPT, Nicotinamide phosphoribosyltransferase; NNMT, Nictotinate N-methyltransferase; NR, Nicotinamide riboside; NMN, Nicotinamide mononucleotide; NAAD+, nicotinate adenine dinucleotide; SIRTs, sirtuins. The hatched line delineates the plasma membrane of the cell.

NAD+ is also generated by vitamin B3 salvage pathways from nicotinate by the enzyme nicotinate phosphoribosyltransferase (NAPT) and from nicotinic acid by the enzyme nicotinamide phosphoribosyltransferase (NAMPT) to produce NAD^+^ along with nicotinamide ribonucleotide (NMN) and nicotinamide riboside (NR)^27^ and both these metabolites are strikingly elevated in PEsen cells relative to the controls (Figs 3 and 4, Supplementary Table S2a). The FDRs ranged from <0.06 to <0.11 for the different comparisons (Supplementary Table S2b). NAD+ levels are maintained in PEsen cells relative to the controls (Fig. 3) but NADH is depleted in PEsen cells resulting in a high NAD+/NADH ratio (Fig. 3) as reported recently for cells induced to senesce by DNA damage^7^. However, both NADH depletion and the increased NAD+/NADH ratio were even more striking in both sets of cell cycle arrested controls suggesting that these metabolic changes are not specific to PEsen cells. NR is a newly discovered NAD^+^ precursor that is converted to NMN by specific NR kinases (NRK) and as NAD^+^ levels were not significantly depleted in PEsen cells (Fig. 3) this suggests that the high levels of NMN and NR may be an indication of either the detoxification of quinolinic acid or increased activity of NAMPT (see above) and/or NAPT to maintain NAD^+^ levels. We were unable to detect nicotinate or its downstream metabolite nicotinate ribonucleotide in our study but the downstream metabolism of nicotinate ribonucleotide (Fig. 4) appears to be reduced (see below). Interestingly, nicotinate^23^ but not nicotinamide (Fig. 3) is depleted in the conditioned medium of PEsen cells supporting a role for increase NAPT activity in PEsen cells. However, although intracellular nicotinate was undetectable in any of the experimental groups (Fig. 4), the breakdown product of nicotinate, trigonelline (N′-methylnicotinate), was elevated in PEsen cells (Fig. 3) and although this did not quite reach statistical significance this suggests that nicotinate is being depleted within the PEsen cells as well.

**Figure 5:**
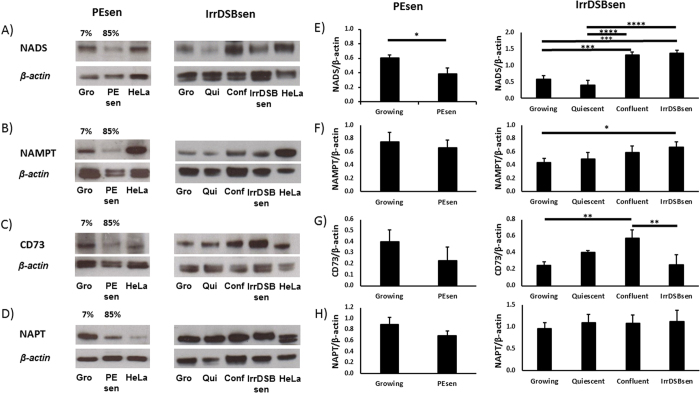
NAD+ salvage pathway enzyme levels in PEsen. Western blots showing the levels of NAD synthetase, NADS, (**A**,**E**), Nicotinamide phosphoribosyltransferase, NAMPT (**B**,**F**), CD73 (**C**,**G**) and Nicotinate phosphoribosyltransferase, NAPT, (**D**,**H**), in PEsen cells versus young growing (Gro) cells (left panels) and IrrDSBsen cells versus growing (Gro), quiescent (Qui) and confluent (Conf) cells. (**A**–**D**) show representative blots of 3 independent experiments that were scanned and quantitated (**E**–**H**). The left and right blots are cropped blots from different experiments and examples of full length blots are illustrated in Supplementary Fig. S5. The histograms represent the mean ratios of the enzyme levels relative to the βactin loading control of the 3 experiments +/− standard deviation. *P < 0.05; **P < 0.01; ***P < 0.001; ****P < 0.0001. A student’s t-test was used for the PEsen (growing vs PEsen) and a one-way ANOVA with Tukey’s post-hoc multiple comparisons test for the IrrDSBsen (growing, quiescent, confluent and IrrDSBsen). The growing cells had completed 23.2–31.1 MPDs, PEsen 62.1–68.3 MPDs and IrrDSBsen 26.1–32.5 MPDs.

To investigate the NAD salvage pathway (Fig. 4) in more detail we conducted western blots of PEsen cells and cells induced to senesce by irreparable DNA damage (IrrDSBsen) as well as all controls (Fig. 5). NAPT was not elevated at all in either IrrDSB or PEsen cells (Fig. 5). We showed that whilst NAMPT was modestly elevated in IrrDSBsen it was also similarly elevated in confluent cells (Fig. 5), whereas NMN and NR were not (Fig. 3) and NAMPT was not elevated in PEsen cells relative to growing controls (Fig. 5). Therefore neither NAMPT nor NAPT appeared responsible for the elevation of NMN or NR. NMN adenylyltransferases (NMNATs) convert NMN to NAD+ via the amidated pathway and also convert nicotinate mononucleotide to deamido NAD+ and thence via NAD+ synthetase to NAD+ via the deamidated pathway (Fig. 4). Unfortunately we were unable to detect NMNATs 1 and 3 on western blots and so were unable to test the levels of NMNATs protein expression. However, nicotinate adenine dinucleotide (NAAD+) was significantly depleted in PEsen cells compared to both growing and confluent cells suggesting that the activity of NMNATs was specifically reduced in senescent cells (Fig. 3). NAD synthetase (NADS) also showed only a modest increase in levels in IrrDSBsen cells and was similarly elevated in confluent cells but was significantly reduced in PEsen (Fig. 5) rendering NADS alterations an unlikely explanation for the high levels of NMN and NR in PEsen cells. Furthermore, the downstream metabolites of nicotinate mononucleotide and NMNAT and the deamidoNAD+/NADS pathways NAAD+, (Fig. 5) and adenosine monophosphate (Supplementary Table S3), respectively, were both reduced in PEsen cells (Fig. 3 – see also below)) arguing against the deamido pathway being responsible for the maintenance of NAD+ in PEsen cells. NR is also imported from the outside of the cell by CD73 (Fig. 4) and is then converted into NMN by NR kinase (NRK) for utilisation in the NAD+ salvage pathway^28^ and as NR was even higher in PEsen cells than NMN we investigated CD73 levels. Western blots of CD73 showed no difference between PEsen or IrrDSBsen cells and the controls (Fig. 5) thus arguing against the hypothesis that increased import of NR was responsible for the elevation of NR and NMN within PEsen cells. However, as we could not detect any extracellular NAD+ or NR in any of the experimental groups it is difficult to rule this out conclusively. Whilst NAD+ is maintained in PEsen cells its conversion to nicotinamide would likely be compromised by the downregulation of the sirtuins^26^. We have confirmed the down regulation of SIRT1 in our PEsen cells (Supplementary Figs S4 and S5). Interestingly, unlike nicotinate, nicotinamide showed only a slight trend for depletion in PEsen media which did not reach statistical significance but level of nicotinamide levels and its breakdown product 1-methylnicotinamide were maintained within PEsen cells and 1-methylnicotinamide increased strikingly in the conditioned medium (Fig. 3).

### Nucleotide catabolism

**Figure 6:**
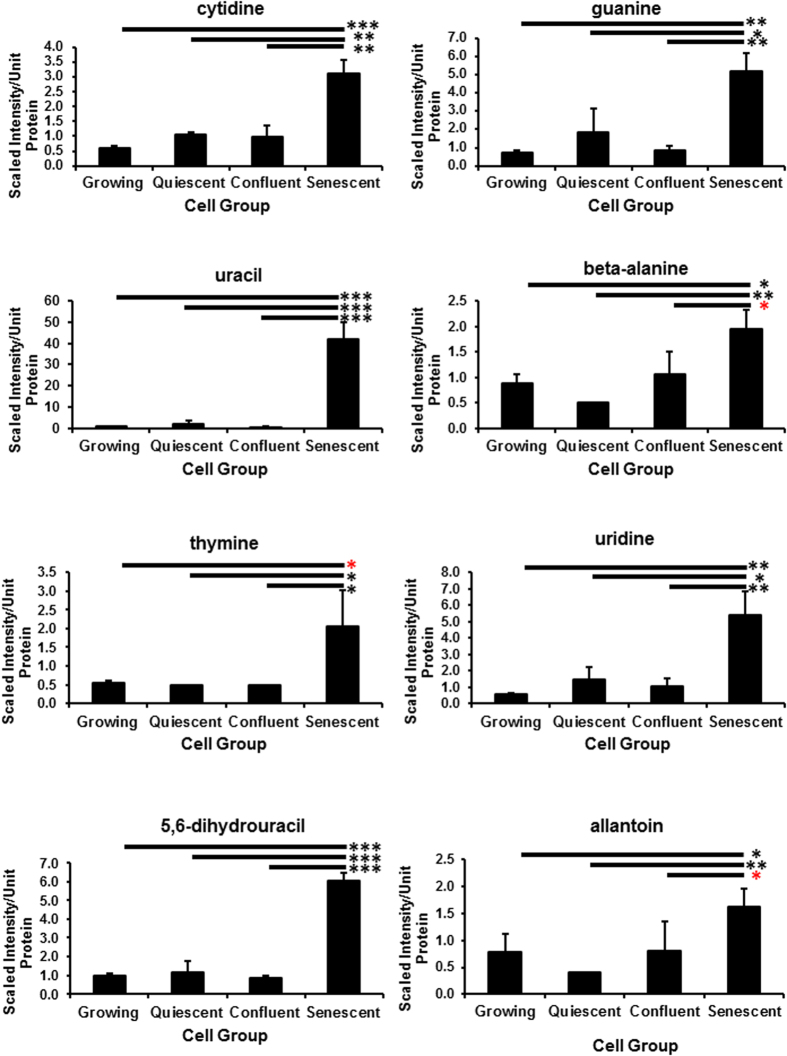
The accumulation of nucleotide catabolites in PEsen cells. The Figure shows the specific accumulation of several nucleotide catabolites in PEsen cells as compared to their growing and growth-arrested controls. Levels of each metabolite normalised to cell protein content +/− standard deviation in growing, quiescent, confluent and PEsen NHOF-1 oral fibroblasts. N = 3 per cell group. The symbols are the same as for Fig. 2.

**Figure 7:**
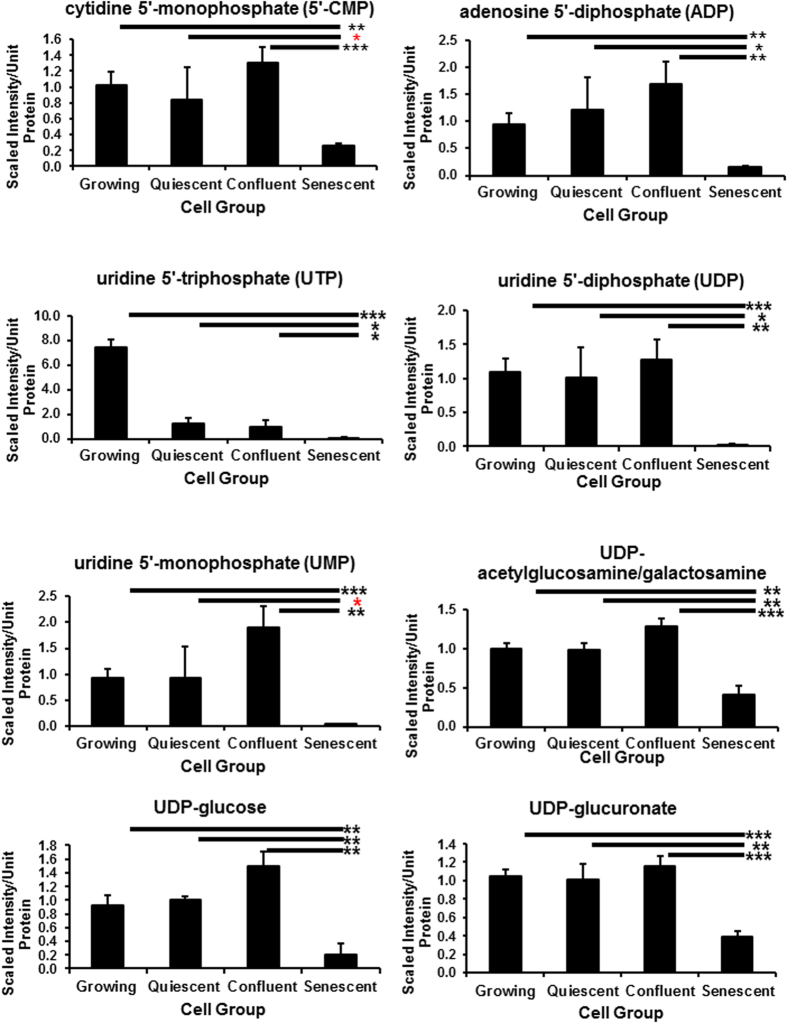
Nucleotide phosphates and their sugars are specifically depleted in PEsen cells. The Figure shows the specific depletion of several nucleotide mono- and di-phosphates and two of their sugars in PEsen cells as compared to their growing and growth-arrested controls. Levels of each metabolite normalised to cell protein content +/− standard deviation in growing, quiescent, confluent and PEsen NHOF-1 oral fibroblasts. N = 3 per cell group. The symbols are the same as for Fig. 2.

Multiple purine and pyrimidine degradation products including 3-uriedoproprionate^23^, cytidine, guanine, guanosine, hypoxanthine^23^, xanthine, allantoin, 5,6-dihydrouracil, uracil, thymine and beta-alanine accumulated in PEsen cells compared to growing counterparts and growth-arrested controls (Fig. 6, Supplementary Table S3a) and the FDRs ranged from <0.06 to <0.11 for the different comparisons (Supplementary Table S3b). In parallel multiple purine and pyrimidine nucleoside mono- and some di- and tri-phosphates were specifically depleted in PEsen cells when compared to the controls, as was adenine and the nucleotide sugars, UDP-acetylglucosamine/galactosamine, UDP-glucose and UDP-glucuronate (Fig. 7, Supplementary Table S3).

### Lipid metabolism

Several lipids were altered within PEsen cells relative to the controls (Supplementary Fig. S6, Supplementary Table S4a) and the FDRs ranged from <0.16 to <0.3 for the different comparisons (Supplementary Table S4b). Arachidonate-derived prostaglandin E2 (PGE2) was significantly elevated in PEsen cells compared to growing and confluent counterparts. Additionally, PEsen cells possessed lower levels of multiple polyunsaturated fatty acids (PUFAs) including docosatrienoate (22:3n3), and dihomo-linoleate (Supplementary Fig. S6, Supplementary Table S4) compared to growing, quiescent and confluent counterparts that may partially be indicative of decreased uptake in PEsen cells as suggested by previously reported elevated levels of these metabolites in corresponding media samples^23^. Similarly to PUFAs, multiple long chain fatty acids including palmitate and stearate were diminished in PEsen cells, but not quiescent counterparts. A similar trend was also observed to less of an extent in confluent cells (Supplementary Fig. S6). Carnitine conjugated lipids including stearoylcarnitine, acetylcarnitine, butylcarnitine and free carnitine were elevated in PEsen cells (Supplementary Fig. S6, Supplementary Table S4) but the last 3 metabolites were also elevated in confluent cells (Supplementary Fig. S7). Related to this pathway, 3-hydroxybutyrate was elevated in PEsen media, although this did not reach statistical significance (Supplementary Fig. S7).

### Membrane lipids

**Figure 8:**
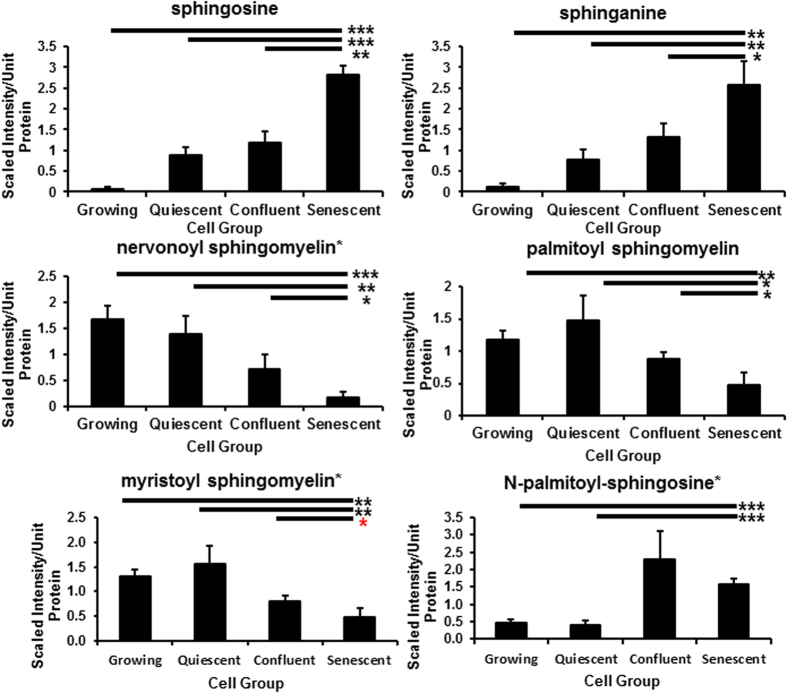
PEsen cells show evidence of membrane degradation. The Figure shows the specific depletion of several sphingomyelins and the accumulation of the membrane breakdown products sphingosine and sphingonine in PEsen cells as compared to their growing and growth-arrested controls. Levels of each metabolite normalised to cell protein content +/− standard deviation in growing, quiescent, confluent and PEsen NHOF-1 oral fibroblasts. N = 3 per cell group. The symbols are the same as for Fig. 2.

The phospholipid catabolite glycerol 3-phosphate was specifically elevated in PEsen cells compared to the controls (Supplementary Table S4) and this trend was also observed in the PEsen media samples as previously reported^23^. In addition to phospholipids, multiple sphingolipid metabolites including sphinganine and sphingosine were specifically elevated in PEsen cells compared to the growing and growth-arrested controls and several sphingolipids, including nervonoyl sphingomyelin. However, N-palmitoyl-sphingosine accumulated to an even greater extent in confluent cells and so the specificity of this result to PEsen is uncertain (Fig. 8, Supplementary Table S4). Palmitoyl sphingomyelin and myristoyl sphingomyelin were correspondingly depleted (Fig. 8, Supplementary Table S4). Further evidence for membrane degradation in PEsen cells is provided by the accumulation of multiple lysolipids in these cells when compared to their growing and growth arrested controls; these included 1-palmitoylglycerophosphoinositol, 1-stearoylglycerophosphoinositol, 1- oleoylglycerophosphoinositol and 1-stearoylglycerophosphoserine (Supplementary Table S4). 1-stearoylglycerol (1-monstearin) also accumulated (Supplementary Table S4) but in contrast, palmitoyl- palmitoyl glycerophosphocholine was depleted (Supplementary Table S4).

### Amino acids and amino acid derivatives

Several amino acids accumulated in PEsen cells when compared to growing and growth-arrested controls, including alanine, creatinine, creatine phosphate and several N-acetylated or -methylated amino acids, whereas other N-acetylated amino acids were depleted (Supplementary Fig. S8, Supplementary Table S5a) and the FDRs ranged from <0.09 to <0.23 for the different comparisons (Supplementary Table S5b). In particular, the N-acetylated amino acids, N-acetyl-asparagine, N-acetylaspartate (NAA) and N-acetylhistidine accumulated specifically in PEsen cells and N-acetylglutamate was specifically depleted (Supplementary Table S5) The methylated amino acid N-methylglutamate accumulated in PEsen cells (Supplementary Table S5).

### Miscellaneous metabolites

Several other metabolites of uncertain significance specifically accumulated in PEsen cells when compared with both the growing and growth-arrested controls (Supplementary Fig. S9, Supplementary Table S6a); these included a xenobiotic (presumably derived from the foetal bovine serum) methyl glucopyranoside (alpha and beta), a neurotransmitter (acetylcholine), an aminosugar (N-acetyl-glucosamine 1-phosphate) and a co-factor/vitamin (phosphopantetheine). The FDRs ranged from <0.03 to <0.05 for the different comparisons (Supplementary Table S6b).

## Discussion

As PEsen intracellular metabolism had not been studied previously in detail we conducted an unbiased metabolic screen of these cells to identify metabolites that were altered when compared not only to young growing cells but also to cells growth-arrested by two independent methods and we report here only metabolites that were specific to senescence. These alterations were still very numerous and although we have yet to conduct flux experiments to identify the rate limiting steps in the various metabolic pathways but with this caveat our data suggests that senescent cells activate pathways that may aid their survival against a background of considerable cellular dysfunction.

We previously reported that PEsen cells show evidence of increased glycolysis and pentose phosphate pathway metabolism^23^. This data was consistent with energy metabolism being diverted away from the TCA cycle in established PEsen cells to avoid further damage to the cell and reduce oxidative damage^23^ especially at the mitochondria^7^,^24^. Consistent with this hypothesis, multiple GGAAs including gamma-glutamylglutamate and gamma-glutamylglutamine^23^ together with cytsteinylglycine were diminished in all three conditions compared to growing cells but his was more marked in PEsen cells. The enzyme gamma-glutamyl transferase (GGT) catalyses the transfer of a gamma-glutamyl moiety of glutathione to an acceptor (an amino acid) and releases cysteinylglycine that can provide cysteine for *de novo* glutathione synthesis. The reduced gamma-glutamyl amino acid levels in PEsen cells could arise from decreased import and catabolism but 5-oxoproline levels did not differ between PEsen cells and the controls. Therefore, reduced cysteinylglycine levels (particularly in PEsen cells) may suggest a decline in GGT activity and/or expression. We previously reported that PEsen cells exhibited a strong trend for reduced levels of cysteine compared to growing, confluent, and quiescent counterparts^23^. This finding may reflect decreased synthesis, considering cystathionine also showed a slight trend for reduction in these cells, or may arise from decreased uptake as suggested by high cysteine levels in the media reported previously^23^. PEsen cells exhibited significantly higher levels of the tripeptide ophthalmate which is an analogue of glutathione in which cysteine has been replaced by 2-aminobutyrate. Cysteine-glutathione disulfide (an internal marker of reactive oxygen species), and cysteine sulfinic acid (generated from the irreversible oxidation of cysteine), which are often indicative of cysteine depletion, were both elevated in PEsen cells and as reported previously, in their media^23^. Cysteine is the rate limiting metabolite for glutathione synthesis and therefore may highlight a difference in utilization and redox homeostasis between conditions. Indeed, diminished levels of GSSG and, as reported previously to a lesser extent, reduced glutathione^23^ in PEsen cells may highlight a limited pool of total cellular glutathione for free radical detoxification. Additionally, PEsen cells possessed detectable levels of S-lactoylglutathione. S-lactoylglutathione is often generated from the hemithioacetal adduct of methylglyoxal and glutathione by the enzyme glyoxylase I and may reflect glutathione consumption for the detoxification of methylglyoxal. Methylglyoxal can arise from lipid peroxidation, threonine catabolism, and non-enzymatic phosphate elimination from glyceraldehyde 3-phosphate and can be associated with inflammation and cell death^29^. Indeed, the lipid peroxidation products 13-HODE^+^9-HODE and 4-hydroxy-nonenal-glutathione (a surrogate for 4-hydroxynonenal) accumulated in PEsen cells, although they also accumulated in confluent cells as well. Finally, the non-significant trend for low levels of alpha-tocopherol in PEsen cells compared to the controls may suggest antioxidant depletion to restore redox homeostasis but this did not reach statistical significance. In addition, we have previously reported an increase in metabolites of the pentose phosphate pathway (PPP)^23^ and this may reflect glucose 6-phosphate shuttling to the PPP to support glutathione detoxification and NADPH regeneration. NADPH is elevated in all the cell cycle arrest conditions examined and whilst this change was not specific to PEsen it may participate in the overall PEsen phenotype. NADPH and the PPP are upregulated in conditions of low stress by p53^30^ that are symptomatic of senescence generated by irreparable DNA damage and PEsen^7^. Taken together, the changes in energy metabolism and redox homeostasis are consistent with the low levels of p53 and p21^WAF^ that are the features of senescent cells established by DNA damage^30^. These new results along with those recently reported^23^ are also consistent with the low level of oxidative damage present in senescent fibroblasts bearing high levels of DNA damage foci rescued from early passage oral submuocus fibrosis cultures^25^ and with the anti-oxidant status of low levels of p53^30^.

Our results also suggest that the kynurenine *de novo* pathway and/or the salvage pathway may have a role in the generation of NAD^+^ to meet the energy demands of PEsen cells in the absence of effective oxidative phosphorylation and this is consistent with the decline in TCA metabolism. However, NADH levels were not maintained in PEsen cells or indeed the cell cycle arrested controls arguing against this hypothesis. We have previously reported that kynurenine is elevated both intracellularly and extracellularly in PEsen fibroblasts compared to controls^23^ and kynurenine can maintain NAD+ levels in cells through the *de novo* pathway from tryptophan. NAD+ levels are maintained in PEsen cells but not NADH levels suggesting that NAD+ levels may contribute to PEsen biology by a mechanism other than energy production. NAD+ is also a co-enzyme that in particular, regulates the family of deacetylases known as the sirtuins (SIRTs) that have multiple anti-senescence functions^27^, and can mediate the anti-ageing effects of caloric restriction^27^.

The main sources of NAD+ within the cell are the salvage pathways from the two forms of vitamin B3, namely nicotinate and nicotinamide. Nicotinate can be converted to NAD+ from nicotinate mononucleotide involving the enzymes NMNAT and NADS via the deamidated route^31^. However, extracellular nicotinate was specifically depleted in PEsen cells as reported previously^23^ and intracellular nicotinate was undetectable, nicotinate mononucleotide, nicotinate riboside and deamido NAD+ as well as NMNAT 1 and 3 were all undetectable in this study. Whilst the inability to detect these metabolites and enzymes should be treated with caution, the NMNAT downstream metabolite NAAD+ was detectable and shown to be significantly downregulated in PEsen cells. NADS was detectable in all experimental groups by western blotting as was the upstream NAPT and whilst the latter was not specifically altered in PEsen cells NADS was specifically downregulated in PEsen cells as was its downstream metabolite, adenosine monophosphate, suggesting that activity though the deamidated pathway was, if anything, reduced. The alternative salvage pathway from vitamin B3 involves the production of NMN from nicotinamide by the NAMPT enzyme which would produce NMN and then NAD+. We observed a striking and specific upregulation of the downstream metabolite of NAMPT, NMN which would suggest an increase in NAMPT activity, the established rate-limiting step for NAD+ production in mammalian cells. NAMPT activity is normally proportional to its protein and transcript levels but we found only a modest increase in NAMPT protein in IrrDSBsen cells that was also observed in confluent cells, whilst in PEsen cells NAMPT levels slightly declined. NR can be generated extracellularly from NMN and/or NAD+ by CD73 and transported into the cell where it is converted to NMN by nicotinamide riboside kinase and thence to NAD+ by NMNAT^28^. However, when we examined the levels of CD73 we found no increase in the former in senescent cells and the downstream metabolite of NMNAT via the deamidated pathway from nicotinate, NAAD+, was reduced.

The maintenance of NAD+, nicotinamide and in PEsen cells is consistent with the retention of some SIRT activity in PEsen cells, although several enzymes can contribute to the production of nicotinamide from NAD+^27^ and we have confirmed that SIRT1 is downregulated in our PEsen cells arguing that NAD+ levels are maintained in PEsen cells by other mechanisms. However, reduced SIRT levels could explain the accumulation of NR and NMN in PEsen cells, although reduced NMNAT activity through the amidated salvage pathway (Fig. 4) cannot be ruled out as a contributing factor at this stage. Interestingly, despite the fact that SIRTs are largely absent from PEsen cells nicotinamide levels are sustained both inside and outside PEsen cells. In addition the downstream breakdown product of nicotinamide, 1-methylnicotinamide, is sustained within the cells and specifically accumulates in PEsen conditioned medium, suggesting increased uptake and turnover of nicotinamide. Alternatively, nicotinamide could be synthesised from NAD+ in the absence of SIRTs by poly(ADPribosyl) polymerase which is known to be an upstream regulator of p53 in PEsen and DNA double strand break-induced senescence^32^. However, the latter mechanism would require that NAD+ was sustained by mechanisms other than the *de novo* and salvage pathways. Although our analysis of NAD+ metabolism is by no means complete, our data is consistent with the hypothesis that PEsen cells sustain nicotinamide and NAD+ levels by as yet undetermined mechanisms to compensate for the loss of SIRT and NMNAT activity which in turn, may explain the accumulation of NMN and NR.

The accumulation of multiple purine and pyrimidine degradation products may contribute to and be indicative of altered redox homeostasis (discussed above) as the generation of xanthine is accompanied by the production of hydrogen peroxide, while allantoin is produced through non-enzymatic oxidation in human cells. Purine and pyrimidine nucleoside mono- and some di- and tri-phosphates were specifically depleted in PEsen cells when compared to the controls, as was adenine. Interestingly, published studies in other cell types suggest that the suppression of nucleotide metabolism underlies the maintenance of OIS^18^,^33^ while the addition of exogenous nucleotides can reverse this phenomenon. However, as far as we are aware the depletion of nucleoside phosphates has not previously been reported in PEsen cells. In our study, these findings may suggest increased catabolism of nucleic acids as opposed to synthesis considering no significant differences were observed between growing and PEsen cells in the nucleotide synthesis intermediates orotate, orotidine, and N-carbamoylaspartate. Nucleotide sugars act as donors for glycosyltransferases and thus their depletion may indicate an increase in the activity of these enzymes in PEsen cells and consequently an increase in O-glycosylation.

We observed elevated levels of PGE2 in PEsen cells which have been reported to both accompany senescence and promote features of the phenotype^34^,^35^. Similarly, PGE2 has been reported to inhibit the growth of quiescent fibroblasts stimulated with serum and growth factors. Differences in lipid metabolism may be indicative of altered hydrolysis, uptake, or oxidation as elevated levels of carnitine conjugated lipids including stearoylcarnitine may reflect increased lipid transport into the mitochondria for oxidation. Indeed, it has been demonstrated that increased fatty acid oxidation results in an unexpectedly high rate of basal oxygen consumption in cells that have undergone OIS^21^ but this has not previously been reported for PEsen. In our study, high levels of acetylcarnitine, which facilitates the movement of acetyl CoA into the matrices of the mitochondria during the oxidation of fatty acids and in confluent and PEsen cells may be indicative of β-oxidation and this was not seen in quiescent cells. In support of this hypothesis, acetylcarnitine and butylcarnitine are often indicative of odd and even chain fatty acid oxidation, respectively. Furthermore, high levels of free carnitine in confluent and PEsen cells may support increased lipid transport into the mitochondria, while high levels of the ketone body 3-hydroxybutyrate, often generated from excess acetyl CoA is a marker of mitochondrial β-oxidation^36^ showed a trend for elevation in PEsen media. Taken together, these data support increased membrane peroxidation in PEsen cells, although it should be noted that elevated acetylcarnitine, butylcarnitine and free carnitine were also seen in confluent cells and PGE2 was elevated in quiescent cells suggesting that these metabolic changes may only be a non-specific part of the PEsen phenotype.

The phospholipid catabolite glycerol 3-phosphate was specifically elevated in PEsen cells and may highlight increased membrane degradation that may provide lipid substrates for the maintenance of energy metabolism and this is consistent with other reports in the literature^37^. In addition to phospholipids, multiple sphingolipid metabolites including sphinganine and sphingosine were specifically elevated in PEsen cells compared to the growing and growth-arrested controls and several sphingolipids, including nervonoyl sphingomyelin, palmitoyl sphingomyelin and myristoyl sphingomyelin were depleted Sphingolipids can mechanically stabilize the plasma membrane lipid bilayer and contribute to signalling events regulating cell survival and apoptosis^38^. N-palmitoyl-sphingosine is a ceramide which accumulates in PEsen cells when compared to growing and quiescent controls and ceramide levels accumulate as cells enter senescence^39^,^40^,^41^. However, in our hands N-palmitoyl-sphingosine accumulated to an even greater extent in confluent cells and so the specificity of this result to PEsen is uncertain. Further evidence for membrane degradation in PEsen cells was provided by the accumulation of multiple lysolipids in these cells, although palmitoyl- palmitoyl glycerophosphocholine was depleted.

Both creatine and creatine phosphate were elevated in PEsen cells. Creatine can serve as a phosphate energy store and this may therefore reflect a difference in energy storage and/or availability in these cells and this increased phosphate store may support cell survival. Creatine metabolites may potentially arise from decreased energy utilization as opposed to generation in confluent and PEsen cells, although this was more marked in PEsen cells. Thus, differences in creatine metabolism in PEsen cells may highlight altered energy storage and utilization in these cells. Alanine increased in PEsen cells and could either be an indication of increased oxidative stress and/or increased glycolysis through the alanine-glucose cycle^42^ (see above). N-acetylation of amino acids can play an important part in the stability and localisation of proteins and so the accumulation of N-acetyl-asparagine, N-acetylaspartate (NAA) and N-acetylhistidine and the depletion of N-acetylglutamate may reflect changes in the levels or intracellular locations of certain proteins but this needs further investigation. The intracellular accumulation of NAA in PEsen fibroblasts is surprising as it is normally only expressed in neurones but is similar to the surprising accumulation of another neurotransmitter, acetylcholine. The methylation of proteins has mainly been studied in histones but the accumulation of N-methylglutamate in PEsen cells (supplementary Table S5) could reflect increased activity of methyltransferases involved in methionine metabolism and would be consistent with the depletion of the S-adenosylmethione (SAM) metabolite S-adenosylhomocysteine (SAH) in the same cells (see above).

We have recently reported that the extracellular senescence metabolome (ESM) of cultured human PEsen fibroblasts shows considerable overlap with that of human serum ageing biomarkers^23^ and irreparable DNA double strand break-induced senescence^23^. In addition the accumulation of the ESM metabolites glycerophosphorylcholine and citrate is reversed in certain mouse models of longevity^23^. However, analysis of the effect of ageing on the intracellular metabolome has so far been confined to the liver and muscle of rodents^43^ but there is considerable evidence for alterations in energy metabolism and redox homeostasis, as both tissues accumulate glucose with age in analogous fashion to PEsen cells. Liver tissue also accumulates glycerol 3-phosphate with age but paradoxically accumulates gamma glutamyl-leucine, alpha tocopherol, cysteine, reduced glutathione and depletes opthalmate. Muscle tissue accumulates glycerophosphorylcholine^23^ and depletes uridine 5′monophosphate analogous to PEsen cells but there are several metabolites that accumulate in the aged mouse muscle that are not seen in PEsen fibroblasts and perhaps this is not surprising as muscle and liver are not dominated by fibroblasts. Further work will be required to established the PEsen metabolome of muscle and liver cells *in vitro*. There are also differences between liver and muscle in methionine metabolism^43^,^44^.

The long-lived Ames Dwarf mouse has been reported to sustain better mitochondrial function and shows reduced insulin signalling and atypical methionine metabolism^45^. Ames Dwarf mice mount an increased oxidative defence in the liver and show increased levels of SAM and its SAH and largely maintain cysteine and reduced glutathione levels, especially when fed low levels of methionine in the diet^44^ while SAM and SAH were specifically depleted in PEsen cells; Ames Dwarf mice also showed reduced levels of hypotaurine, multiple gamma glutamyl amino acids, opthalmate and noropthalmate compared to control mice^44^ and these metabolites were generally elevated in PEsen cells (this study and ref. 23) consistent with Ames Dwarf mouse liver cells using these antioxidant pathways to prevent senescence initiation. However, Ames Dwarf mouse liver tissue paradoxically showed elevated S-lactoylglutathione in common with PEsen cells when fed low, but not high, levels of methionine; cystathione is also depleted under both sets of conditions^44^ and in PEsen cells. Thus there are pathways in common between PEsen human fibroblasts and the long-lived Ames Dwarf mouse as regards oxidative defence. The manner in which the oxidative stress pathways are regulated in PEsen cells and the Ames Dwarf liver tissue is generally consistent with the Ames Dwarf mouse antagonising ageing by upregulating multiple mechanisms of oxidative defence that are generally reduced in established PEsen cells but nevertheless are sufficient to enable the latter to survive.

In addition to their association with ageing several of the metabolic pathways described above are associated with other diseases and processes. Kynurenine can activate the immune system that could perhaps lead to the clearance of PEsen cells *in vivo*. Kynurenine can also be neuroprotective if metabolised to kynurenic acid but also has the potential to mediate deleterious effects as its metabolism in the liver can lead to the generation of metabolites such as 3-hydroxykynurenine that can cross the blood-brain barrier and contribute to neurodegeneration^46^,^47^,^48^. In addition, kynurenine has been reported to be associated with cardiovascular disease (CVD)^49^,^50^ and to be reduced upon treatment with omega-3 polyunsaturated fatty acids, which in turn are thought to reduce the risk of CVDs^51^. In addition, both NMN and NR have antioxidant, antiageing and neuroprotective effects^27^,^52^ but paradoxically were strikingly elevated in PEsen cells in parallel with sustained levels of NAD+, suggesting that PEsen cells *in vitro* and perhaps *in vivo* possess properties normally associated with anti-ageing strategies. NAMPT levels are reduced in the neuronal stem/progenitor cells of the hippocampus with ageing^52^ and genetic or pharmacological inhibition of NAMPT results in neuronal stem/progenitor cell depletion and impairment but here it is reported that NAMPT levels were if anything slightly elevated in PEsen cells and both nicotinamide and NAD+ levels were maintained in PEsen cells. Sustaining NAD+ levels may allow PEsen cells to survive for long periods *in vitro*^12^ and perhaps *in vivo* should they evade the immune system^3^,^4^, even in the presence of a persistent DNA damage response^13^.

## Conclusions

Although the metabolic profile of established PEsen cells is superficially complex there are two conclusions that are consistent with a large amount of the data. Firstly, PEsen cells appear to activate the same mechanisms that delay the initiation of senescence; a shift from oxidative phosphorylation towards glycolysis and the pentose phosphate pathway to reduce the production of ROS^23^ and an upregulation of pathways involved in redox homeostasis to ameliorate the effects of hydrogen peroxide. Secondly, other alterations such as the maintenance of NAD+ and nicotinamide levels, nucleotide catabolism, membrane breakdown and alterations in creatine metabolism could be interpreted as a strategy the senescent cells adopt to promote cell survival and generate energy and the building blocks for cell growth in the absence of functional mitochondria and oxidative phosphorylation. Our results also suggest caution in the use of supplements such as nicotinamide and antioxidants, as whilst there is evidence that these compounds may delay cellular senescence and age-related diseases, they are unlikely to eliminate existing PEsen cells and may actually aid their survival. Lastly, senescent cells are known to be generally resistant to apoptosis^1^ and recently targeting apoptotic pathways has achieved some success in selectively eliminating senescent cells and producing ‘senolytic drugs’^14^,^53^. Therefore, some of the senescence-specific pro-survival alterations in the metabolic pathways described here may also serve as targets for the future development of such drugs.

## Experimental Procedures

### Cell Culture

The NHOF-1 oral fibroblast line its characterisation and the culture methods have been described previously^23^,^25^. Briefly, NHOF-1 fibroblasts were grown in Dulbecco’s Modified Eagles Medium containing 10% vol/vol foetal bovine serum (FBS) in an atmosphere of 10% CO_2_/90% air and subcultured once weekly at a density of 1 × 10^5^ cells per 9 cm plate to prohibit confluence. Mean population doublings (MPDs) were calculated as described previously^54^. SA-βGal activity was assessed using the senescence detection kit from Biovision^25^. Quiescent control cultures were allowed to remain confluent or in 0.1% vol/vol FBS for 4 days before analysis and the medium changed every day. The PEsen and growing cultures were also medium changed every day. The NHOF-1 cells were defined as PEsen by numerous markers^23^,^25^ and by the extension of replicative lifespan following the retroviral transduction of the catalytic component of telomerase (James and Parkinson – unpublished data). In some experiments senescent cells were generated by the introduction of irreparable DNA damage and this was achieved by irradiating NHOF-1 cells in suspension with 20 Gy of ionising radiation and then culturing them for 20 days, as described previously^23^.

### Cell pellet preparation for metabolomics analysis

Cell pellets were collected from growing, quiescent, confluent and PEsen NHOF-1 cells essentially as described^55^. Briefly, the cells were rinsed with 0.02% EDTA and incubated with 0.1% trypsin and 0.01% EDTA until they were detached, the trypsin/EDTA solution was neutralised by adding 2 × volume of growth medium containing FBS and the cells were centrifuged at 300xG for 3 minutes after an aliquot was taken for counting. The cells were then washed in calcium- and magnesium-free phosphate-buffered saline and centrifuged again before removing the supernatant and freezing the pellet as above. The amount of protein in each cell pellet was measured by the Bradford Assay.

### Metabolomic analysis, normalisation and data presentation as scaled intensity

The details of the metabolomics analysis have been published previously, including sample preparation, instrumentation, conditions for mass spectrometry (liquid chromatography/tandem mass spectrometry in positive and negative ion modes, and gas chromatography/mass spectrometry), peak data reduction, and assignment of peaks to known chemical entities by comparison to metabolite library entries of purified standards, has been previously described^23^. Briefly, for analysis, the median signal intensity of a given biochemical was determined across all sample groups. This median was subsequently used to scale individual samples to a median of 1 for the group. A minimum value was assigned when a biochemical was not detected in an individual sample (this was rare). This data is graphically presented as scaled intensity and is thus a measure of the relative level of each metabolite. The cell pellet scaled intensities were then further normalised to account for the variation in biomass between different experimental groups and expressed as scaled intensity per unit protein. For further details of the metabolomics analyses see supplementary experimental procedures.

### Western blotting

Western blotting was carried out as described previously^56^ using the following antibodies: Anti NAD Synthetase Rabbit monoclonal Abcam Cat # Ab171942; Anti NAPT rabbit polyclonal Abcam Cat # Ab127699; Anti visfatin (NAMPT) mouse monoclonal Abcam Cat # Ab71505; Anti-CD73 rabbit polyclonal Abcam Cat # Ab137595. HeLa cell extracts were used as positive controls.

### Statistical Analysis

A two-sample *T*-test was used to identify biochemicals that differed significantly between experimental groups and in some instances one way analysis of variance (ANOVA) and Tukeys *post hoc* test were used. Pathways were assigned for each metabolite, allowing examination of overrepresented pathways. Principal component analysis was utilized to determine the metabolites that differed between the four experimental groups^57^, and integrated hierarchical clustering analysis was used to classify them by groupings of metabolites^58^. Welch’s *T* tests were carried out on log-transformed data to compare relative biochemical concentrations between experimental groups. False discovery rate (FDR) was estimated using *q* values to account for multiple comparisons.

## Additional Information

**How to cite this article**: James, E. L. *et al*. Replicatively senescent human fibroblasts reveal a distinct intracellular metabolic profile with alterations in NAD+ and nicotinamide metabolism. *Sci. Rep.* **6**, 38489; doi: 10.1038/srep38489 (2016).

**Publisher’s note:** Springer Nature remains neutral with regard to jurisdictional claims in published maps and institutional affiliations.

## Supplementary Material

Supplementary Information
