# Supplementary material for: Replicatively senescent human fibroblasts reveal a distinct intracellular metabolic profile with alterations in NAD+ and nicotinamide metabolism
Source: Sci Rep. 2016 Dec 7;6:38489. doi: 10.1038/srep38489 (PMC5141431; doi:10.1038/srep38489)
Supplement: Supplementary Information [file srep38489-s1.pdf]

Replicatively senescent human fibroblasts reveal a distinct intracellular metabolic profile with alterations in NAD+ and nicotinamide metabolism.

Emma L. James[1], James A.E. Lane[1], Ryan D. Michalek[2], Edward D. Karoly[2] and E. Kenneth Parkinson[1,3]

[1]Centre for Clinical & Diagnostic Oral Sciences, Institute of Dentistry, Barts and the London School of Medicine and Dentistry, Queen Mary University of London, Turner Street, London E1 2AD, UK

[2] Metabolon, Inc. 617 Davis Drive, Suite 400, Durham, NC 27713

[3]Correspondence to: Eric Kenneth Parkinson, Centre for Clinical & Diagnostic Oral Sciences, Blizard Institute, 4 Newark Street, London E1 2AT UK, Phone: 44-(0)207-882-7185 Fax: 44(0)207-882-7137, Email: e.k.parkinson@qmul.ac.uk

Supplementary Information

Experimental Procedures

Global Metabolomics

Sample Preparation for Global Metabolomics

Samples were stored at –80°C until processed. Sample preparation was carried out as described previously [1] at Metabolon, Inc. Briefly, recovery standards were added prior to the first step in the extraction process for quality control purposes. To remove protein, dissociate small molecules bound to protein or trapped in the precipitated protein matrix, and to recover chemically diverse metabolites, proteins were precipitated with methanol under vigorous shaking for 2 min (Glen Mills Genogrinder 2000) followed by centrifugation. The resulting extract was divided into five fractions: one for analysis by ultra-high performance liquid chromatography-tandem mass spectrometry (UPLC-MS/MS; positive ionization), one for analysis by UPLC-MS/MS (negative ionization), one for the UPLC-MS/MS polar platform (negative ionization), one for analysis by gas chromatography–mass spectrometry (GC-MS), and one sample was reserved for backup.

Three types of controls were analyzed in concert with the experimental samples: samples generated from a pool of human plasma extensively characterized by Metabolon, Inc. or generated from a small portion of each experimental sample of interest served as technical replicate throughout the data set; extracted water samples served as process blanks; and a cocktail of standards spiked into every analyzed sample allowed instrument performance monitoring. Instrument variability was determined by calculating the median relative standard deviation (RSD) for the standards that were added to each sample prior to injection into the mass spectrometers (median RSD typically = 4-6%; n ≥ 30 standards). Overall process variability was determined by calculating the median RSD for all endogenous metabolites (i.e., non-instrument standards) present in 100% of the pooled human plasma or client matrix samples (median RSD = 8-12%; n = several hundred metabolites). Experimental samples and controls were randomized across the platform run.

Mass Spectrometry Analysis

Non-targeted MS analysis was performed at Metabolon, Inc. Extracts were subjected to either GC-MS [2] or UPLC-MS/MS [1]. The chromatography was standardized and, once the method was validated no further changes were made. As part of Metabolon's general practice, all columns were purchased from a single manufacturer's lot at the outset of experiments. All solvents were similarly purchased in bulk from a single manufacturer's lot in sufficient quantity to complete all related experiments. For each sample, vacuum-dried samples were dissolved in injection solvent containing eight or more injection standards at fixed concentrations, depending on the platform. The internal standards were used both to assure injection and chromatographic consistency. Instruments were tuned and calibrated for mass resolution and mass accuracy daily.

The UPLC-MS/MS platform utilized a Waters Acquity UPLC with Waters UPLC BEH C18-2.1×100 mm, 1.7 µm columns and a Thermo Scientific Q-Exactive high resolution/accurate mass spectrometer interfaced with a heated electrospray ionization (HESI-II) source and Orbitrap mass analyzer operated at 35,000 mass resolution. The sample extract was dried then reconstituted in acidic or basic LC-compatible solvents, each of which contained 8 or more injection standards at fixed concentrations to ensure injection and chromatographic consistency. One aliquot was analyzed using acidic, positive ion-optimized conditions and the other using basic, negative ion-optimized conditions in two independent injections using separate dedicated columns (Waters UPLC BEH C18-2.1x100 mm, 1.7 µm). Extracts reconstituted in acidic conditions were gradient eluted using water and methanol containing 0.1% formic acid, while the basic extracts, which also used water/methanol, contained 6.5mM ammonium bicarbonate. A third aliquot was analyzed via negative ionization following elution from a HILIC column (Waters UPLC BEH Amide 2.1x150 mm, 1.7 µm) using a gradient consisting of water and acetonitrile with 10mM Ammonium Formate. The MS analysis alternated between MS and data-dependent MS^2 scans using dynamic exclusion, and the scan range was from 80-1000 *m/z*.

The samples destined for analysis by GC-MS were dried under vacuum desiccation for a minimum of 18 h prior to being derivatized under dried nitrogen using bistrimethyl-silyltrifluoroacetamide. Derivatized samples were separated on a 5% diphenyl / 95% dimethyl polysiloxane fused silica column (20 m x 0.18 mm ID; 0.18 um film thickness) with helium as carrier gas and a temperature ramp from 60° to 340°C in a 17.5 min period. All samples were analyzed on a Thermo-Finnigan Trace DSQ fast-scanning single-quadrupole MS using electron impact ionization (EI) and operated at unit mass resolving power. The scan range was from 50–750 m/z.

Compound Identification, Quantification, and Data Curation

Metabolites were identified by automated comparison of the ion features in the experimental samples to a reference library of chemical standard entries that included retention time, molecular weight (m/z), preferred adducts, and in-source fragments as well as associated MS spectra and curated by visual inspection for quality control using software developed at Metabolon [3]. Identification of known chemical entities is based on comparison to metabolomic library entries of purified standards. Commercially available purified standard compounds have been acquired and registered into LIMS for distribution to both the UPLC-MS/MS and GC-MS platforms for determination of their detectable characteristics. Peaks were quantified using area-under-the-curve. Raw area counts for each metabolite in each sample were normalized to correct for variation resulting from instrument inter-day tuning differences by the median value for each run-day, therefore, setting the medians to 1.0 for each run. This preserved variation between samples but allowed metabolites of widely different raw peak areas to be compared on a similar graphical scale. Missing values were imputed with the observed minimum after normalization.

Characterisation of NHOF-1 PEsen cells and controls

Senescence-associated β galactosidase (SA-βGal) activity, Ki67 (cycling cells), large 53BP1 foci (irreparable DNA strand breaks) were used as markers of cellular senescence [4, 5]. Immunofluorescence images were quantified using the Image J program as described previously [4].

Western blotting

Western blotting was carried out as described previously [6] using the following antibody: Anti SIRT1 Mouse monoclonal Abcam Cat # Ab110304. HeLa cell extracts were used as positive controls.

Supplementary Table S1a Glutathione, methionine, cysteine and gamma-glutamyl metabolism

Pathway	Metabolite	PEsen v Growing Control	PEsen v Quiescent Control	PEsen v Confluent Control
Methionine, Cysteine, SAM and Taurine Metabolism	cysteine sulfinic acid	0.01	0.008	0.01
Methionine, Cysteine, SAM and Taurine Metabolism	hypotaurine	0.0007	0.001	0.002
Methionine, Cysteine, SAM and Taurine Metabolism	methionine sulfoxide	0.0009	0.003	0.09
Methionine, Cysteine, SAM and Taurine Metabolism	S-adenosylhomocysteine (SAH)	0.03	0.08	0.03
Methionine, Cysteine, SAM and Taurine Metabolism	S-adenosylmethionine (SAM)	0.003	0.04	0.11
Gamma-glutamyl Amino Acid	gamma-glutamylglutamate	0.0002	0.03	0.04
Gamma-glutamyl Amino Acid	gamma-glutamylphenylalanine	0.002	0.002	0.006
Gamma-glutamyl Amino Acid	gamma-glutamylalanine	0.001	0.0003	0.1
Glutathione Metabolism	noropthalamate	0.03	0.03	0.03
Glutathione Metabolism	opthalamate	0.004	0.0002	0.03
Glutathione Metabolism	S-lactoylglutathione	0.01	0.01	0.01
Glutathione Metabolism	glutathione (GSSG)	0.008	0,03	0.01
Lysine Metabolism	pipecolate	0.008	0.02	0.02

P values were determined by the unpaired two sample T test. N = 3.

Supplementary Table S1b Glutathione, methionine, cysteine and gamma-glutamyl metabolism

Pathway	Metabolite	PEsen v Growing Control	PEsen v Quiescent Control	PEsen v Confluent Control
Methionine, Cysteine, SAM and Taurine Metabolism	cysteine sulfinic acid	0.09	0.006	0.03
Methionine, Cysteine, SAM and Taurine Metabolism	hypotaurine	0.02	0.009	0.006
Methionine, Cysteine, SAM and Taurine Metabolism	methionine sulfoxide	0.23	0.02	0.09
Methionine, Cysteine, SAM and Taurine Metabolism	S-adenosylhomocysteine (SAH)	0.25	0.04	0.07
Methionine, Cysteine, SAM and Taurine Metabolism	S-adenosylmethionine (SAM)	0.01	0.06	0.097
Gamma-glutamyl Amino Acid	gamma-glutamylglutamate	0.01	0.02	0.04
Gamma-glutamyl Amino Acid	gamma-glutamylphenylalanine	0.13	0.008	0.01
Gamma-glutamyl Amino Acid	gamma-glutamylalanine	0.03	0.01	0.11
Glutathione Metabolism	noropthalamate	0.27	0.007	0.01
Glutathione Metabolism	opthalamate	0.02	0.02	0.07
Glutathione Metabolism	S-lactoylglutathione	ND	0.02	0.04
Glutathione Metabolism	glutathione (GSSG)	0.17	0.02	0.03
Lysine Metabolism	pipecolate	0.06	0.02	0.04

False discovery rates as determined by the Welch's *t*-test and indicated by q values of less than 0.1 are considered most significant and are highlighted in red when accumulated and green for depleted. ND = not determined.

Supplementary Table S2a Tryptophan and NAD+ metabolism

Pathway	Metabolite	PEsen v Growing Control	PEsen v Quiescent Control	PEsen v Confluent Control
Tryptophan Metabolism	kynurenine	0.0003	0.0001	0.0008
Nicotinate and Nicotinamide Metabolism	nicotinamide adenine dincleotide (NAD+)	0.17	0.41	0.17
Nicotinate and Nicotinamide Metabolism	nicotinamide ribonucleotide (NMN)	3×10^{-5}	0.0002	3×10^{-5}
Nicotinate and Nicotinamide Metabolism	nicotinamide riboside	0.0006	0.0006	00006
Nicotinate and Nicotinamide Metabolism	nicotinamide adenine dinucleotide reduced (NADH)	0.005	0.08	0.005

P values were determined by the unpaired two sample T test. N = 3.

Supplementary Table S2b Tryptophan and NAD+ metabolism

Pathway	Metabolite	PEsen v Growing Control	PEsen v Quiescent Control	PEsen v Confluent Control
Tryptophan Metabolism	kynurenine	0.02	0.008	0.01
Nicotinate and Nicotinamide Metabolism	nicotinamide adenine dincleotide (NAD+)	0.11	0.12	0.11
Nicotinate and Nicotinamide Metabolism	nicotinamide ribonucleotide (NMN)	0.05	0.02	0.0008
Nicotinate and Nicotinamide Metabolism	nicotinamide riboside	0.11	0.01	0.006
Nicotinate and Nicotinamide Metabolism	nicotinamide adenine dinucleotide reduced (NADH)	0.02	0.06	0.06

False discovery rates as determined by the Welch's *t*-test and indicated by q values of less than 0.1 are considered most significant and are highlighted in red when accumulated and green for depleted.

Supplementary Table S3a Nucleotide Pathways

Pathway	Metabolite	PEsen v Growing Control	PEsen v Quiescent Control	PEsen v Confluent Control
Pyrimidine Metabolism, Uracil containing	uracil	0.0008	0.001	0.0008
Pyrimidine Metabolism, Uracil containing	uridine	0.004	0.001	0.007
Pyrimidine Metabolism, Uracil containing	uridine 5'-diphosphate (UDP)	0.0005	0.02	0.002
Pyrimidine Metabolism, Uracil containing	uridine 5'-monphosphate (UMP)	0.0008	0.06	0.001
Pyrimidine Metabolism, Uracil containing	uridine 5'-triphosphate (UTP)	2.69×10^{-5}	0.02	0.03
Pyrimidine Metabolism, Cytidine containing	cytidine	0.0006	0.001	0.003
Pyrimidine Metabolism, Cytidine containing	cytidine 5'-monophosphate (5'-CMP)	0.002	0.07	0.0009
Pyrimidine Metabolism, Thymine containing	thymine	0.05	0.04	0.04
Purine Metabolism, Adenine containing	adenine	6.76×10^{-5}	0.01	0.01
Purine Metabolism, Adenine containing	adenosine 5'-diphosphate (ADP)	0.003	0.04	0.004
Purine Metabolism, Adenine containing	adenosine 3',5'-cyclic monophosphate (cAMP)	0.03	0.12	0.003

Pathway	Metabolite	PEsen v Growing Control	PEsen v Quiescent Control	PEsen v Confluent Control
Purine Metabolism, Guanine containing	guanine	0.001	0.02	0.002
Purine Metabolism, Guanine containing	guanosine	0.001	0.03	0.004
Purine Metabolism, Guanine containing	guanosine 5'-monophosphate (5'-GMP)	0.03	0.12	0.002
Purine Metabolism, (Hypo)xanthine/Inosine containing	hypoxanthine	9.56×10^{-5}	0.03	0.0003
Purine Metabolism, (Hypo)Xanthine/Inosine containing	allantoin	0.04	0.003	0.09
Purine Metabolism, (Hypo)Xanthine/Inosine containing	inosine 5'-monophosphate (IMP)	0.01	0.13	0.02
Nucleotide Sugar	UDP-acetylglucosamine/galactosamine	0.001	0.002	0.0006
Nucleotide Sugar	UDP-glucose	0.005	0.001	0.001
Nucleotide Sugar	UDP-glucuronate	0.0003	0.004	0.0004

P values were determined by the unpaired two sample T test. N = 3

Supplementary Table S3b Nucleotide Pathways

Pathway	Metabolite	PEsen v Growing Control	PEsen v Quiescent Control	PEsen v Confluent Control
Pyrimidine Metabolism, Uracil containing	uracil	0.13	0.01	0.004
Pyrimidine Metabolism, Uracil containing	uridine	0.06	0.02	0.03
Pyrimidine Metabolism, Uracil containing	uridine 5’-diphosphate (UDP)	0.21	0.006	0.006
Pyrimidine Metabolism, Uracil containing	uridine 5’-monphosphate (UMP)	0.22	0.02	0.004
Pyrimidine Metabolism, Uracil containing	uridine 5’-triphosphate (UTP)	0.02	0.007	0.03
Pyrimidine Metabolism, Cytidine containing	cytidine	0.01	0.006	0.04
Pyrimidine Metabolism, Cytidine containing	cytidine 5'-monophosphate (5'-CMP)	0.17	0.04	0.004
Pyrimidine Metabolism, Thymine containing	thymine	0.09	0.02	0.04
Purine Metabolism, Adenine containing	adenine	0.005	0.02	0.02
Purine Metabolism, Adenine containing	adenosine 5'-diphosphate (ADP)	0.23	0.02	0.005
Purine Metabolism, Adenine containing	adenosine 3',5'-cyclic monophosphate (cAMP)	0.07	0.06	0.01
Purine Metabolism, Adenine containing	adenosine 5'-monophosphate (AMP)	0.21	0.02	0.002

Pathway	Metabolite	PEsen v Growing Control	PEsen v Quiescent Control	PEsen v Confluent Control
**Purine Metabolism, Guanine containing**	**guanine**	**0.09**	**0.04**	**0.006**
**Purine Metabolism, Guanine containing**	**guanosine**	**0.04**	**0.04**	**0.04**
**Purine Metabolism, Guanine containing**	**guanosine 5'-monophosphate (5'-GMP)**	**0.26**	**0.07**	**0.01**
**Purine Metabolism, (Hypo)xanthine/Inosine containing**	**hypoxanthine**	**0.1**	**0.05**	**0.01**
**Purine Metabolism, (Hypo)Xanthine/Inosine containing**	**allantoin**	**0.09**	**0.01**	**0.09**
**Purine Metabolism, (Hypo)Xanthine/Inosine containing**	**inosine 5'-monophosphate (IMP)**	**0.07**	**0.07**	**0.05**
**Nucleotide Sugar**	**UDP-acetylglucosamine/galactosamine**	**0.25**	**0.02**	**0.03**
**Nucleotide Sugar**	**UDP-glucose**	**0.16**	**0.04**	**0.06**
**Nucleotide Sugar**	**UDP-glucuronate**	**0.23**	**0.008**	**0.006**

False discovery rates as determined by the Welch’s *t*-test and indicated by q values of less than 0.1 are considered most significant and are highlighted in red when accumulated and green for depleted.

Supplementary Table S4a Lipid Pathways

Pathway	Metabolite	PEsen v Growing Control	PEsen v Quiescent Control	PEsen v Confluent Control
Lysolipid	1-palmitoylglycerophosphoinositol*	0.001	0.003	0.009
Lysolipid	1-stearoylglycerophosphoinositol	0.0002	0.0009	0.03
Lysolipid	1-stearoylglycerophosphoserine*	0.0007	0.003	0.03
Lysolipid	palmitoyl- palmitoyl glycerophosphocholine	0.001	7.22×10^{-7}	7.39×10^{-6}
Lysolipid	1-oleoylglycerophosphoinositol*	0.009	0.02	0.06
Polyunsaturated Fatty Acid (n3 and n6)	docosatrienoate (22:3n3)	0.004	0.0003	0.02
Polyunsaturated Fatty Acid (n3 and n6)	docosadienoate (22:2n6)	0.03	0.01	0.03
Polyunsaturated Fatty Acid (n3 and n6)	adrenate (22:4n6)	0.003	0.02	0.03
Polyunsaturated Fatty Acid (n3 and n6)	dihomo-linoleate (20:2n6)	0.001	0.03	0.08
Polyunsaturated Fatty Acid (n3 and n6)	dihomo-linolenate (20:3n3 or n6)	0.02	0.02	0.07
Polyunsaturated Fatty Acid (n3 and n6)	docosapentaenoate (n6 DPA; 22:5n6)	0.005	0.003	0.07
Sphingolipid Metabolism	nervonoyl sphingomyelin	0.0007	0.004	0.04
Sphingolipid Metabolism	palmitoyl sphingomyelin	0.005	0.02	0.03
Sphingolipid Metabolism	sphinganine	0.001	0.007	0.03
Sphingolipid Metabolism	sphingosine	3.78×10^{-5}	0.0003	0.001
Sphingolipid Metabolism	myristoyl sphingomyelin*	0.005	0.01	0.07
Phospholipid Metabolism	cytidine 5'-diphosphocholine	0.008	0.03	0.001

Pathway	Metabolite	PEsen v Growing Control	PEsen v Quiescent Control	PEsen v Confluent Control
Phospholipid Metabolism	glycerophosphoinositol	0.001	0.04	0.01
Fatty Acid, Dicarboxylate	2-hydroxyadipate	0.01	0.04	0.01
Fatty Acid and BCAA Metabolism	propionylcarnitine	0.007	0.002	0.04
Fatty Acid metabolism (Acyl Carnitine)	stearoylcarnitine	0.008	0.008	0.01
Monoacyl glycerol	1-stearoylglycerol (1-monostearin)	0.001	0.002	0.02
Glycerolipid Metabolism	glycerol-3-phosphate (G3P)	0.02	0.04	0.02
Long Chain Fatty Acid	eicosenoate (20:1n9 or 11)	0.03	0.03	0.11
Eicosanoid	13,14-dihydro-15-keto-prostaglandin A2	9.6×10^{-5}	0.07	0.0001
Eicosanoid	prostaglandin E2	0.005	0.53	0.02

P values were determined by the unpaired two sample T test. N = 3.

Supplementary Table S4b Lipid Pathways

Pathway	Metabolite	PEsen v Growing Control	PEsen v Quiescent Control	PEsen v Confluent Control
Lysolipid	1-palmitoylglycerophosphoinositol*	0.096	0.03	0.07
Lysolipid	1-stearoylglycerophosphoinositol	0.01	0.01	0.08
Lysolipid	1-stearoylglycerophosphoserine*	0.01	0.16	0.3
Lysolipid	palmitoyl- palmitoyl glycerophosphocholine	0.02	0.005	0.004
Lysolipid	1-oleoylglycerophosphoinositol*	0.08	0.02	0.09
Polyunsaturated Fatty Acid (n3 and n6)	docosatrienoate (22:3n3)	0.05	0.01	0.02
Polyunsaturated Fatty Acid (n3 and n6)	docosadienoate (22:2n6)	0.03	0.01	0.02
Polyunsaturated Fatty Acid (n3 and n6)	adrenate (22:4n6)	0.08	0.01	0.03
Polyunsaturated Fatty Acid (n3 and n6)	dihomo-linoleate (20:2n6)	0.02	0.02	0.04
Polyunsaturated Fatty Acid (n3 and n6)	dihomo-linolenate (20:3n3 or n6)	0.12	0.01	0.06
Polyunsaturated Fatty Acid (n3 and n6)	docosapentaenoate (n6 DPA; 22:5n6)	0.05	0.01	0.05
Sphingolipid Metabolism	nervonoyl sphingomyelin	0.13	0.01	0.04
Sphingolipid Metabolism	palmitoyl sphingomyelin	0.14	0.01	0.07
Sphingolipid Metabolism	sphinganine	0.05	0.01	0.04
Sphingolipid Metabolism	sphingosine	0.02	0.01	0.03
Sphingolipid Metabolism	myristoyl sphingomyelin*	0.13	0.01	0.08
Phospholipid Metabolism	cytidine 5'-diphosphocholine	0.18	0.01	0.006

Pathway	Metabolite	PEsen v Growing Control	PEsen v Quiescent Control	PEsen v Confluent Control
**Phospholipid Metabolism**	**glycerophosphoinositol**	**0.04**	**0.02**	**0.05**
**Fatty Acid, Dicarboxylate**	**2-hydroxyadipate**	**0.02**	**0.02**	**0.01**
**Fatty Acid and BCAA Metabolism**	**propionylcarnitine**	**0.01**	**0.006**	**0.05**
**Fatty Acid metabolism (Acyl Carnitine)**	**stearoylcarnitine**	**ND**	**0.02**	**0.03**
**Monoacyl glycerol**	**1-stearoylglycerol (1-monostearin)**	**0.05**	**0.009**	**0.09**
**Glycerolipid Metabolism**	**glycerol-3-phosphate (G3P)**	**0.07**	**0.02**	**0.01**
**Long Chain Fatty Acid**	**eicosenoate (20:1n9 or 11)**	**0.05**	**0.02**	**0.07**
**Eicosanoid**	**13,14-dihydro-15-keto-prostaglandin A2**	**0.13**	**0.07**	**.007**
**Eicosanoid**	**prostaglandin E2**	**0.02**	**0.14**	**0.04**

False discovery rates as determined by the Welch's *t*-test and indicated by q values of less than 0.1 are considered most significant and are highlighted in red when accumulated and green for depleted. ND = not determined.

Supplementary Table S5a Amino acids and amino acid derivatives.

Pathway	Metabolite	PEsen v Growing Control	PEsen v Quiescent Control	PEsen v Confluent Control
Alanine and Aspartate Metabolism	N-acetylasparagine	0.003	0.002	0.04
Alanine and Aspartate Metabolism	N-acetylaspartate(NAA)	0.04	0.02	0.01
Creatine Metabolism	creatine phosphate	0.005	0.004	0.006
Creatine Metabolism	creatinine	0.002	5.9×10^{-5}	0.004
Glutamate Metabolism	N-methylglutamate	0.003	1.3x10-5	0.04
Glutamate Metabolism	N-acetylglutamate	0.001	0.01	0.02
Leucine, Isoleucine and Valine Metabolism	isobutyrlcarnitine	0.01	0.007	0.01
Tryptophan Metabolism	kynurenine	0.0003	0.0001	0.0008
Histidine Metabolism	N-acetylhistidine	0.006	0.04	0.02

P values were determined by the unpaired two sample T test. N = 3

Supplementary Table S5b Amino acids and amino acid derivatives.

Pathway	Metabolite	PEsen v Growing Control	PEsen v Quiescent Control	PEsen v Confluent Control
Alanine and Aspartate Metabolism	N-acetyl-asparagine	0.007	0.009	0.09
Alanine and Aspartate Metabolism	N-acetylaspartate(NAA)	0.23	0.01	0.03
Creatine Metabolism	creatine phosphate	0.09	0.008	0.01
Creatine Metabolism	creatinine	0.15	0.008	0.04
Glutamate Metabolism	N-methylglutamate	0.007	0.004	0.09
Glutamate Metabolism	N-acetylglutamate	0.01	0.04	0.06
Leucine, Isoleucine and Valine Metabolism	isobutyrlcarnitine	0.12	0.02	0.04
Histidine Metabolism	N-acetylhistidine	0.01	0.03	0.03

False discovery rates as determined by the Welch's *t*-test and indicated by q values of less than 0.1 are considered most significant and are highlighted in red when accumulated and green for depleted.

Supplementary Table S6a Miscellaneous metabolites

Pathway	Metabolite	PEsen v Growing Control	PEsen v Quiescent Control	PEsen v Confluent Control
Neurotransmitter	acetylcholine	0.01	0.009	0.01
Food Component/Plant	methyl glucopyranoside (alpha and beta)	0.001	0.002	0.001
Aminosugar Metabolism	N-acetyl-glucosamine 1-phosphate	0.03	0.03	0.03
Pantothenate and CoA Metabolism	phosphopantetheine	5.74 x 10-5	4.49 x 10-5	4.43 x 10-5

P values were determined by the unpaired two sample T test. N = 3.

Supplementary Table S6b Miscellaneous metabolites

Pathway	Metabolite	PEsen v Growing Control	PEsen v Quiescent Control	PEsen v Confluent Control
Neurotransmitter	acetylcholine	0.05	0.02	0.02
Food Component/Plant	methyl glucopyranoside (alpha and beta)	0.05	0.04	0.03
Aminosugar Metabolism	N-acetyl-glucosamine 1-phosphate	0.02	0.006	0.005
Pantothenate and CoA Metabolism	phosphopantetheine	0.03	0.006	0.03

False discovery rates as determined by the Welch's *t*-test and indicated by q values of less than 0.1 are considered most significant and are highlighted in red when accumulated and green for depleted.

A

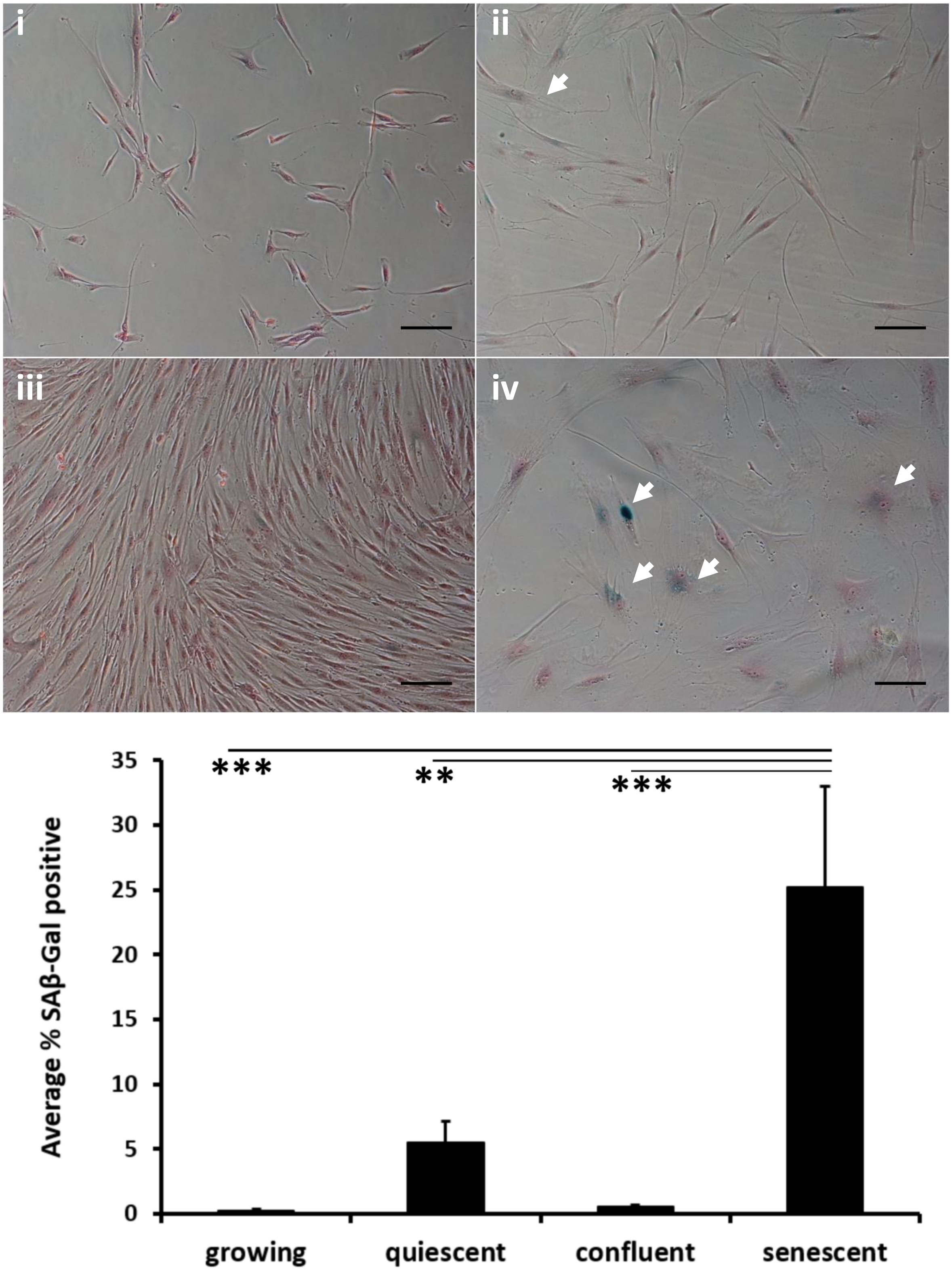
i
ii
iii
iv
***
**
***
Average % SAβ-Gal positive
35
30
25
20
15
10
5
0
growing
quiescent
confluent
senescent

B.

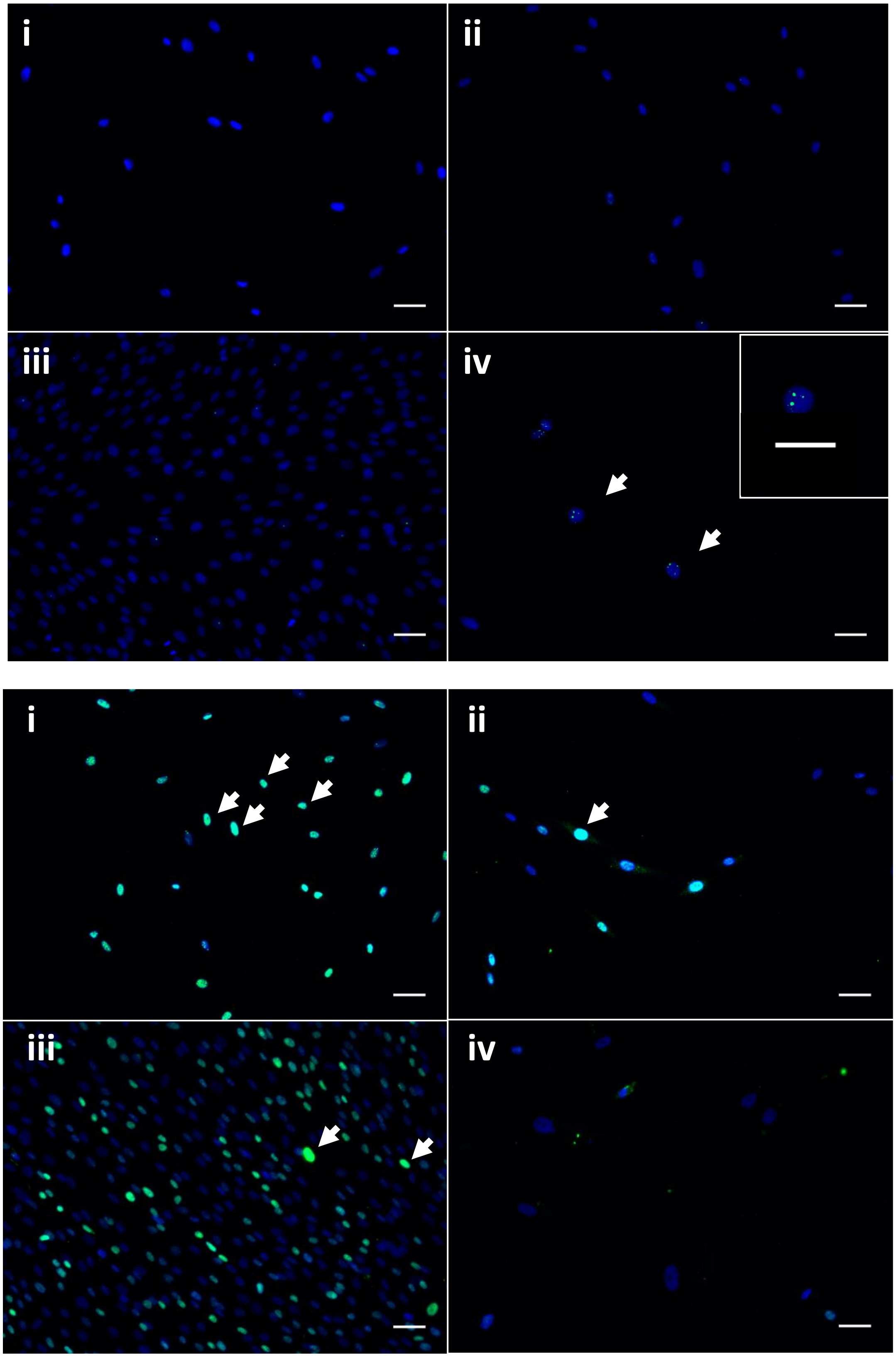

Supplementary Fig.S1 Characterisation of the PEsen and control phenotypes

A. SA-βGal in growing (i), quiescent (ii), confluent (iii) and PEsen (iv) cells; ***p<0.001 **p<0.01 1 way ANOVA Tukey's post hoc.

B. (Upper panel) 53BP1 in the same cells; inset high power image showing large nuclear foci.

(Lower panel) Ki67 in the same cells. Symbols in B are the same as for A. Bar = 50µm throughout. Arrows indicate examples of positive cells; blue cytoplasm in A; green or turquoise nuclei in B and C in antibody-labelled cells counterstained with Dapi (blue nuclei in negative cells).

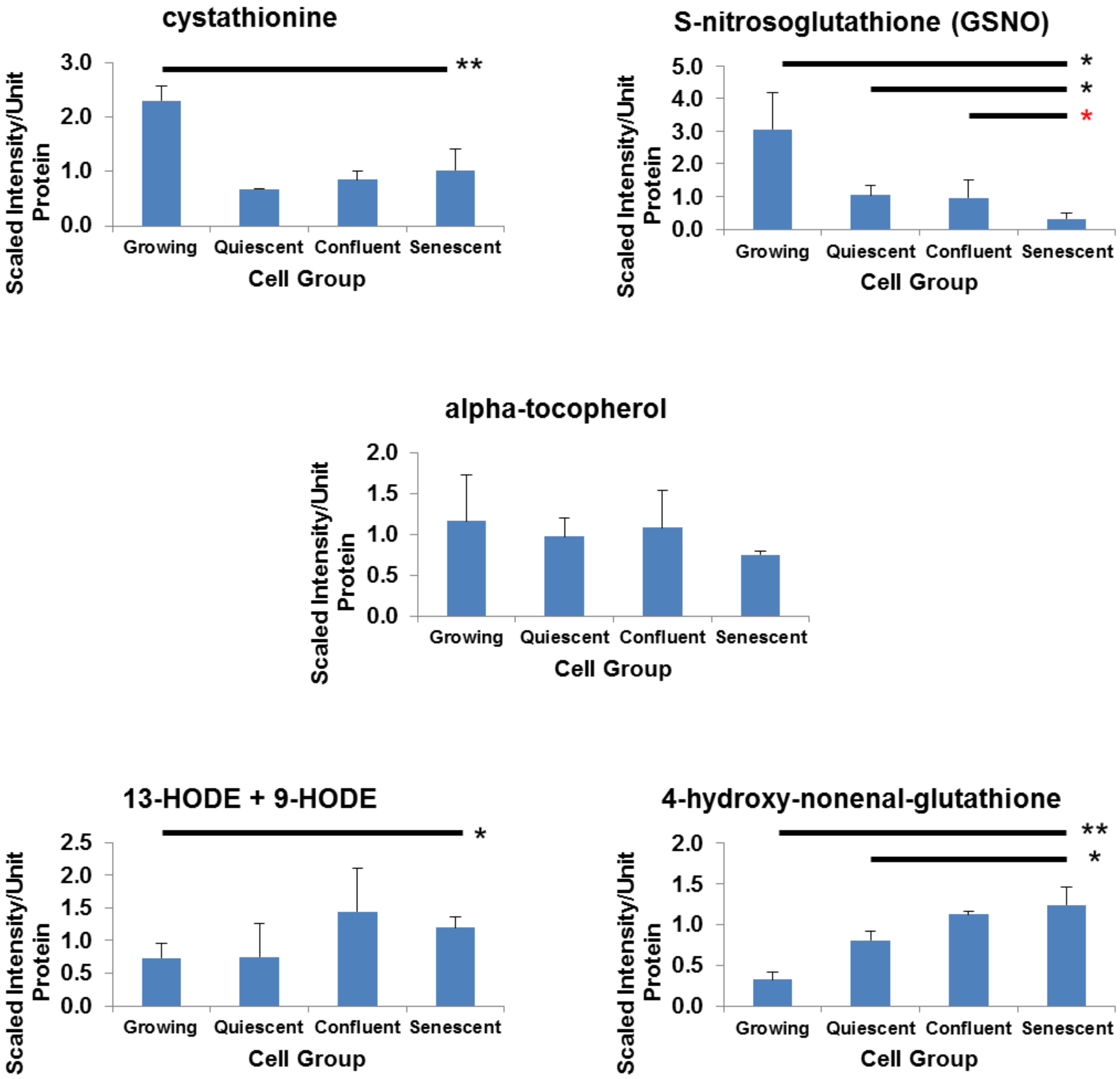

Supplementary Fig. S2 Modulation of intracellular redox metabolites and lipid peroxidation products in PEsen NHOF-1 cells relative to growing, quiescent and confluent cells.

The Figure shows the miscellaneous antioxidants and lipid peroxidation products. Levels of each metabolite normalised to cell protein content +/- standard deviation in growing, quiescent, confluent and PEsen NHOF-1 oral fibroblasts. N = 3 per cell group. The symbols indicate statistically significant results between PEsen cells and the other experimental groups as assessed by unpaired *t*-test and indicate P values as follows: * P > 0.05 <0.1, * P< 0.05, ** P< 0.01, *** P< 0.001.

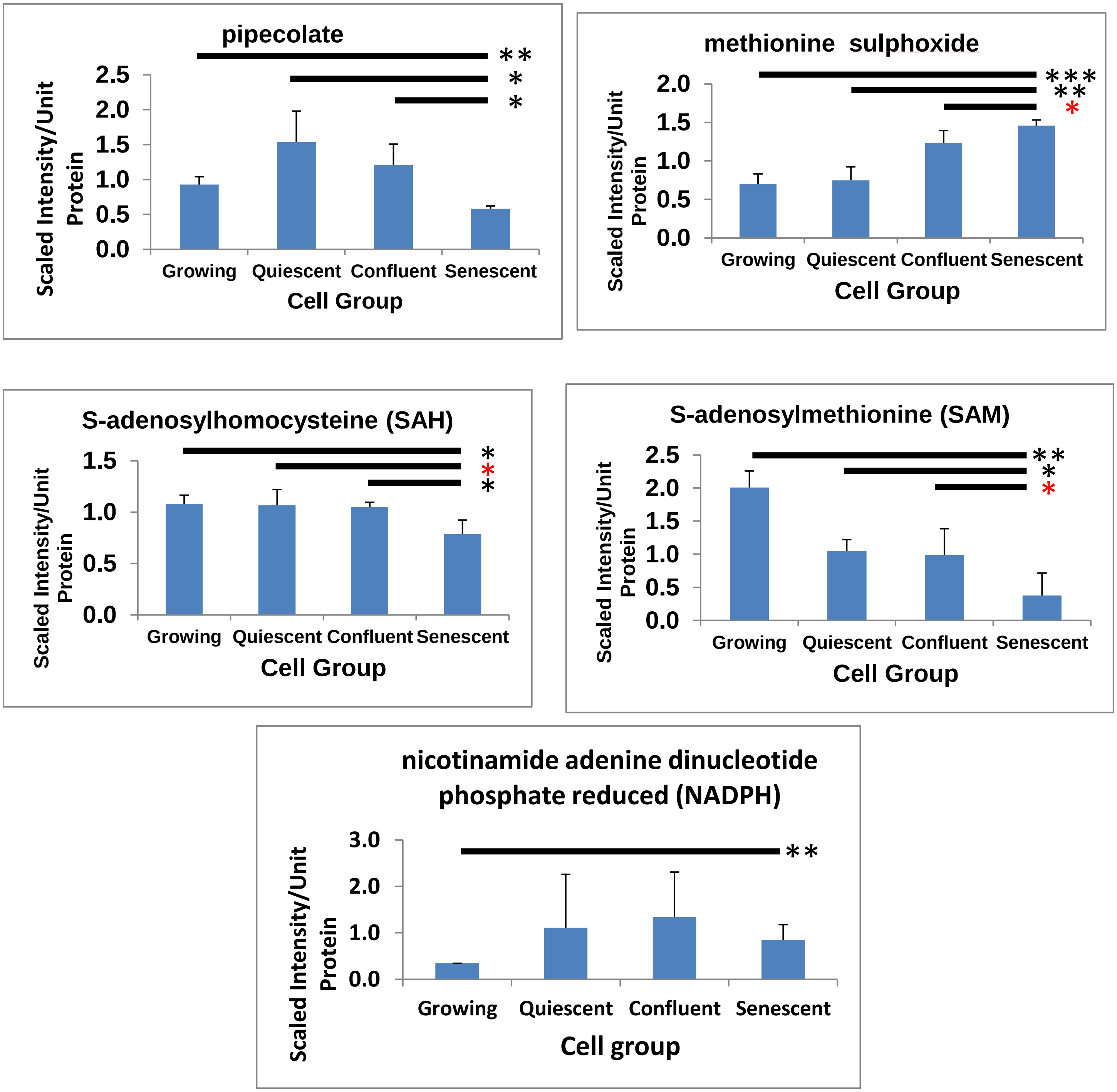

Supplementary Fig. S3 Modulation of intracellular redox metabolites in PEsen NHOF-1 cells relative to growing, quiescent and confluent cells.
The Figure shows the redox homestasis metabolites. The levels of each metabolite were normalised to cell protein content +/- standard deviation in growing, quiescent, confluent and PEsen NHOF-1 oral fibroblasts. N = 3 per cell group. The symbols are the same as for Supplementary Fig. S 2.

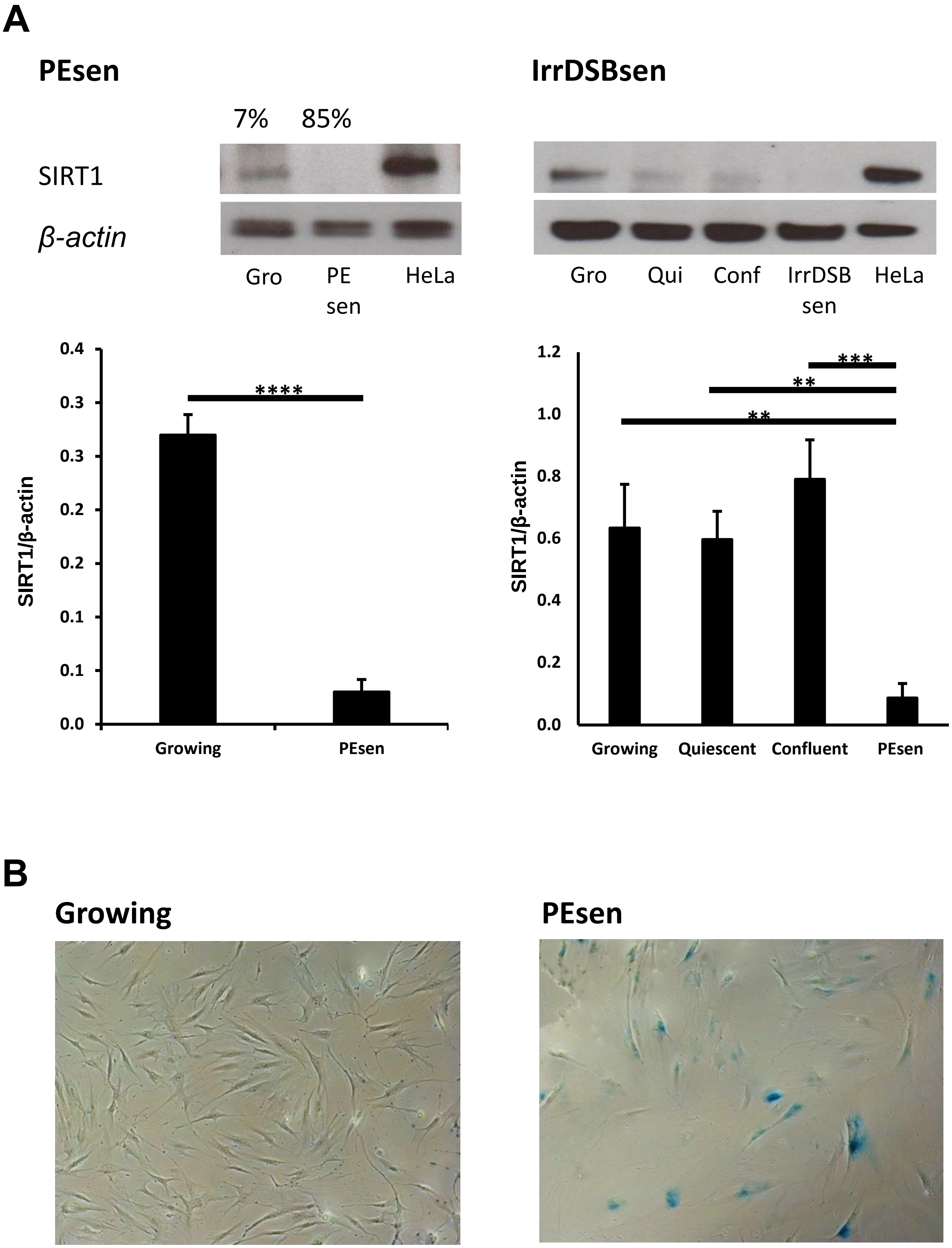

Supplementary Fig. S4 A. The top left panel shows a representative western blot of SIRT1 in PEsen versus growing cells, the top right panel shows a similar blot of growing versus, quiescent, confluent and IrrDSBsen cells and the bottom left and right panels show quantitation of SIRT relative to the βactin loading control from 3 independent experiments +/- standard deviation. The figures above the PEsen and growing lanes indicate the %SA-βGal-positive cells (blue) illustrated in B. The growing cells had completed 23.2-31.1 MPDs and the PEsen cells 62.1-68.3 MPDs and IrrDSBsen 26.1-32.5 MPDs

PEsen

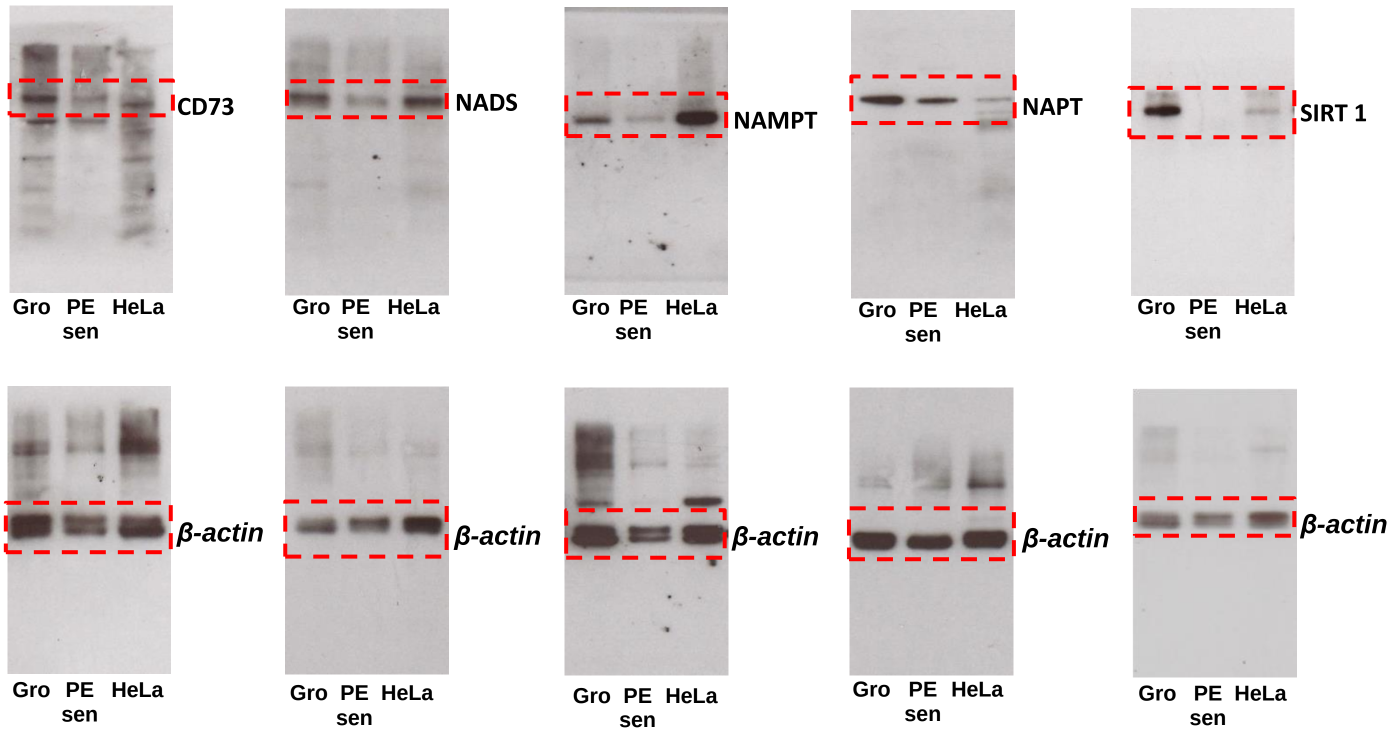

IrrDSBsen

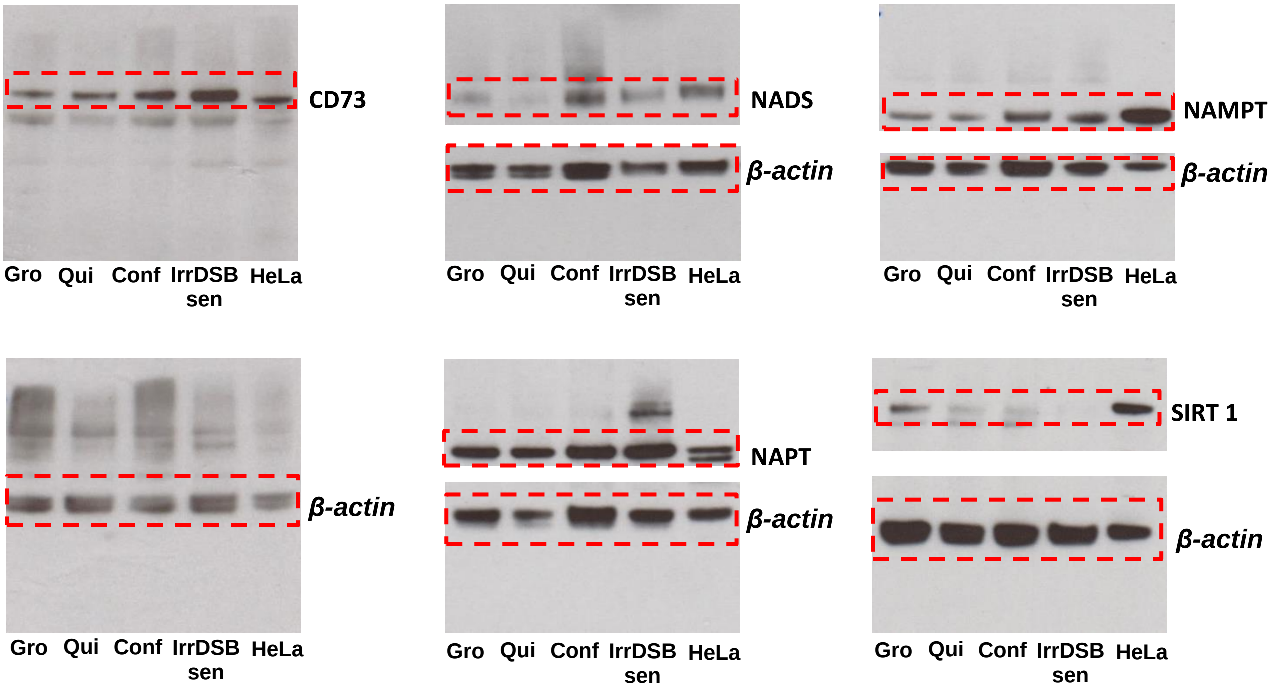

Supplementary Fig. S5 Illustrates the full length western blots of the cropped blots shown in Fig.1 and Supplementary Fig. S4. The hatched red lines delineate the correct sized bands for each protein.

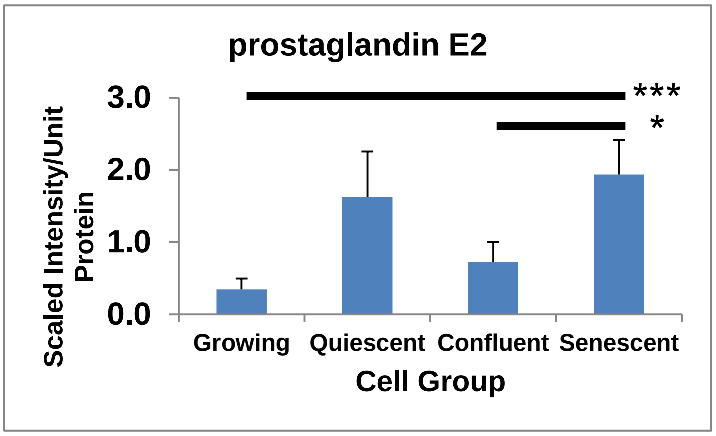
prostaglandin E2
Scaled Intensity/Unit Protein
3.0
2.0
1.0
0.0
***
*
Growing
Quiescent
Confluent
Senescent
Cell Group

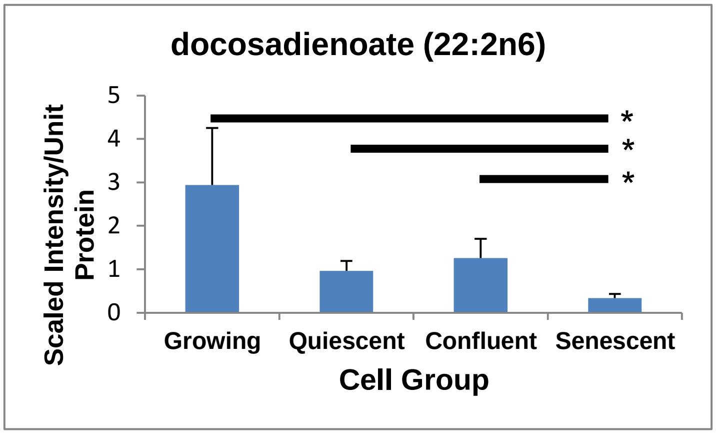
docosadienoate (22:2n6)
Scaled Intensity/Unit Protein
5
4
3
2
1
0
*
*
*
Growing
Quiescent
Confluent
Senescent
Cell Group

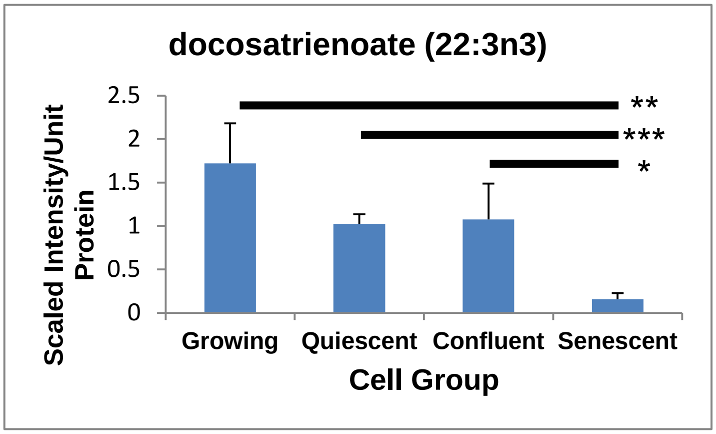
docosatrienoate (22:3n3)
Scaled Intensity/Unit Protein
2.5
2
1.5
1
0.5
0
**
***
*
Growing
Quiescent
Confluent
Senescent
Cell Group

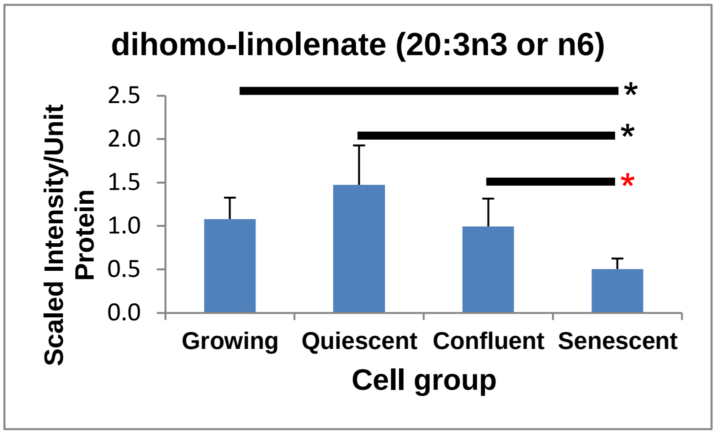
dihomo-linolenate (20:3n3 or n6)
Scaled Intensity/Unit Protein
2.5
2.0
1.5
1.0
0.5
0.0
*
*
*
Growing
Quiescent
Confluent
Senescent
Cell group

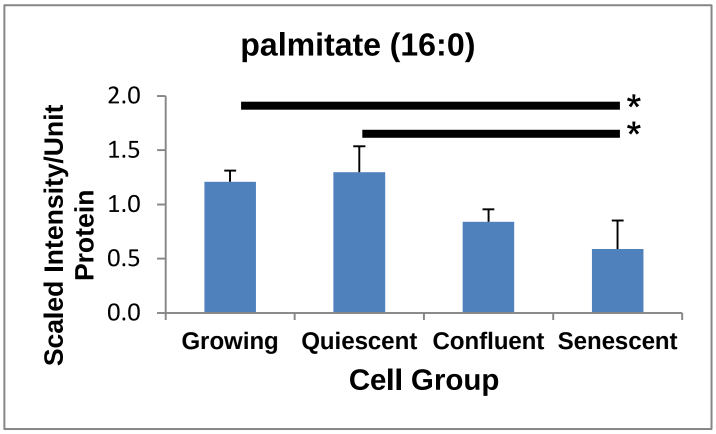
palmitate (16:0)
Scaled Intensity/Unit Protein
2.0
1.5
1.0
0.5
0.0
*
*
Growing
Quiescent
Confluent
Senescent
Cell Group

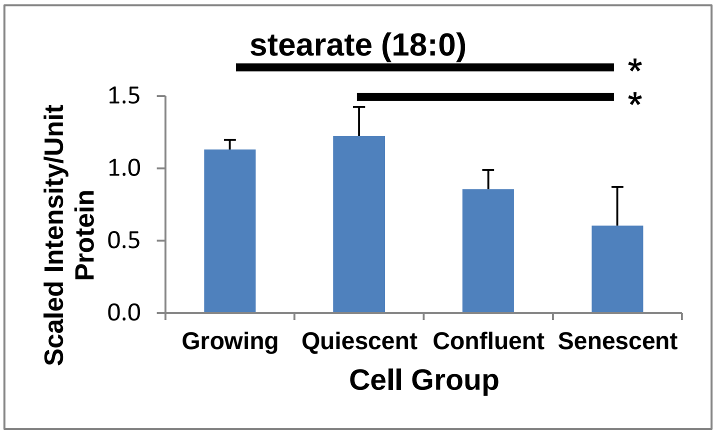
stearate (18:0)
Scaled Intensity/Unit Protein
1.5
1.0
0.5
0.0
*
*
Growing
Quiescent
Confluent
Senescent
Cell Group

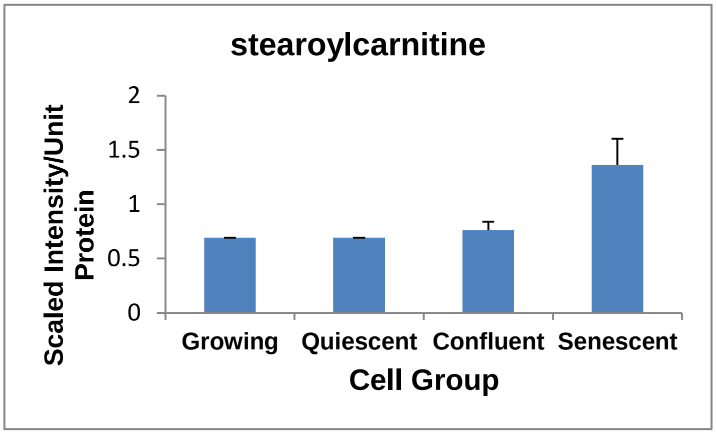
stearoylcarnitine
Scaled Intensity/Unit Protein
2
1.5
1
0.5
0
Growing
Quiescent
Confluent
Senescent
Cell Group

Supplementary Fig. S6 Modulation of intracellular lipids in PEsen NHOF-1 cells relative to growing, quiescent and confluent cells.

The Figure shows the levels of each metabolite normalised to cell protein content +/- standard deviation in growing, quiescent, confluent and PEsen NHOF-1 oral fibroblasts. The symbols are the same as for Supplementary Fig. S2. N = 3 per cell group.

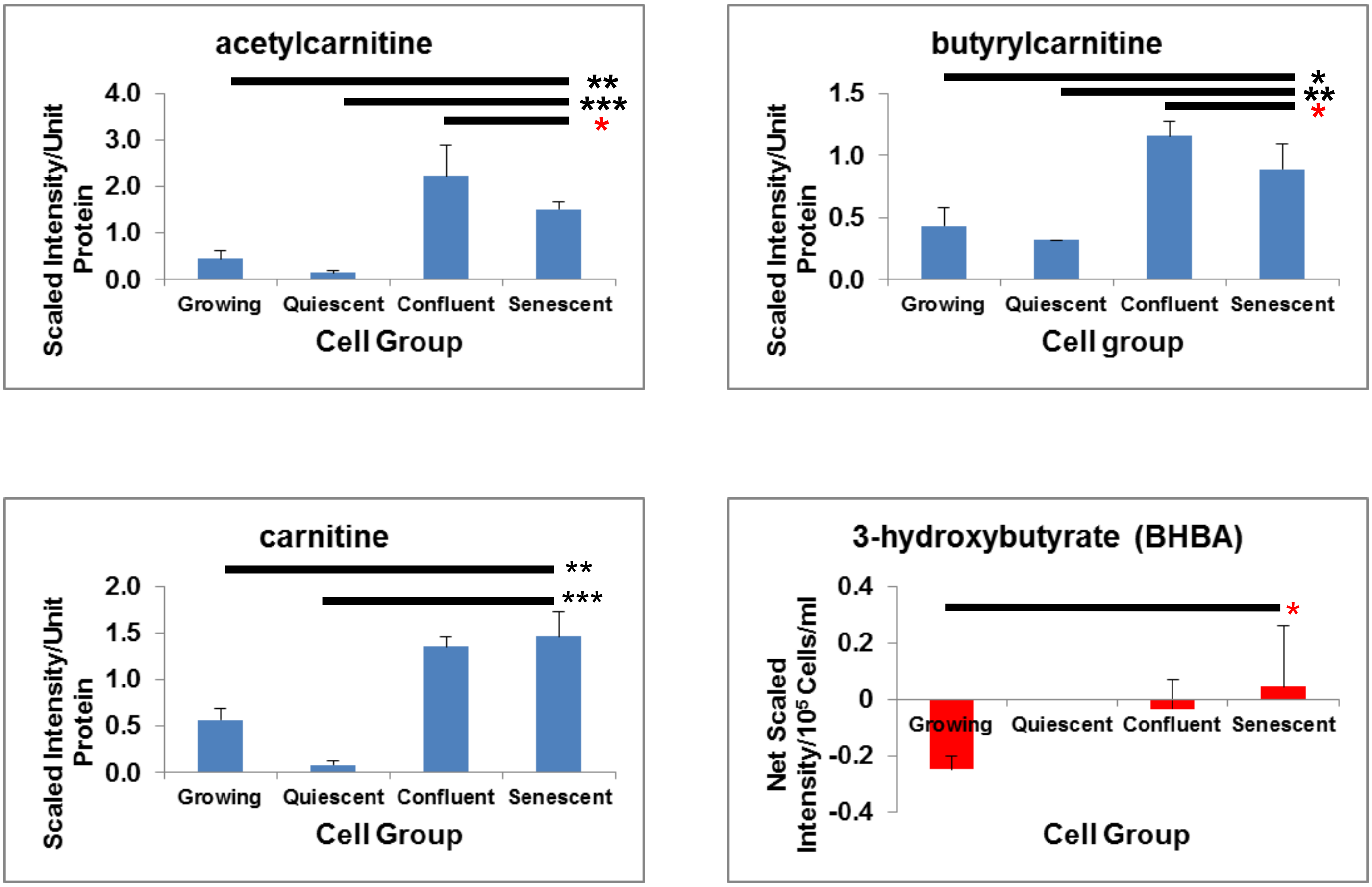

Supplementary Fig. S7 Modulation of intracellular oxidized lipids (blue bars) extracellular ketones (red bars) in PEsen NHOF-1 cells relative to growing, quiescent and confluent cells.
The Figure shows the levels of each metabolite normalised to cell protein content +/- standard deviation (blue) in growing, quiescent, confluent and PEsen NHOF-1 oral fibroblasts or in the medium expressed as net scaled intensity/10^5 cells/ml (red). The symbols are the same as for Supplementary Fig. S2. N = 3 per cell group.

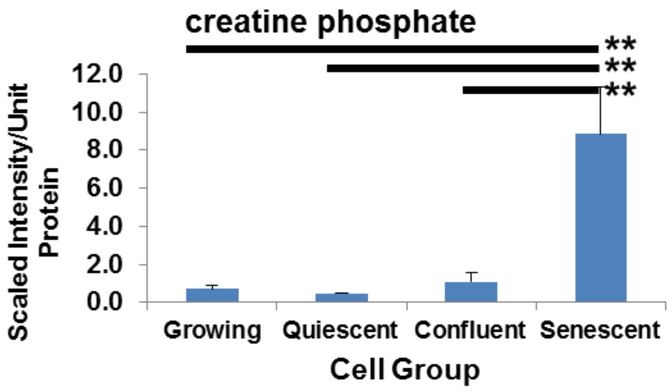
creatine phosphate
**
**
**
Scaled Intensity/Unit Protein
12.0
10.0
8.0
6.0
4.0
2.0
0.0
Growing
Quiescent
Confluent
Senescent
Cell Group

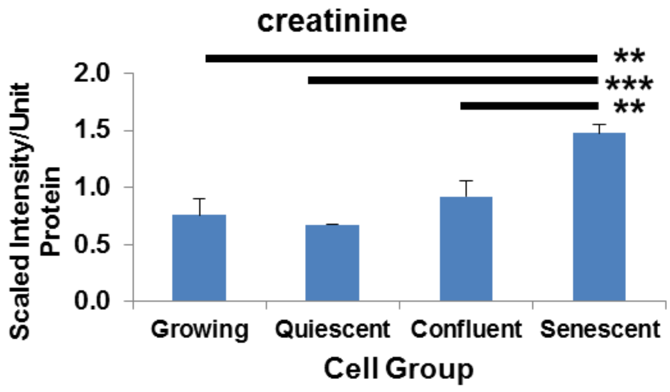
creatinine
**
***
**
Scaled Intensity/Unit Protein
2.0
1.5
1.0
0.5
0.0
Growing
Quiescent
Confluent
Senescent
Cell Group

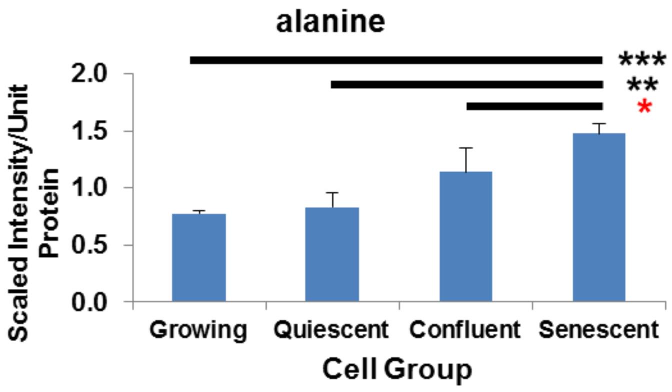
alanine
***
**
*
Scaled Intensity/Unit Protein
2.0
1.5
1.0
0.5
0.0
Growing
Quiescent
Confluent
Senescent
Cell Group

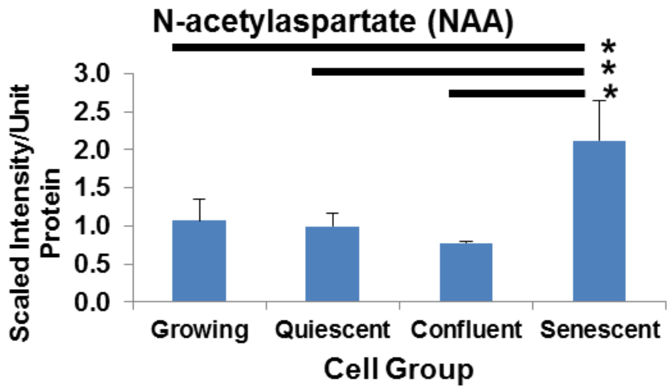
N-acetylaspartate (NAA)
*
*
*
Scaled Intensity/Unit Protein
3.0
2.5
2.0
1.5
1.0
0.5
0.0
Growing
Quiescent
Confluent
Senescent
Cell Group

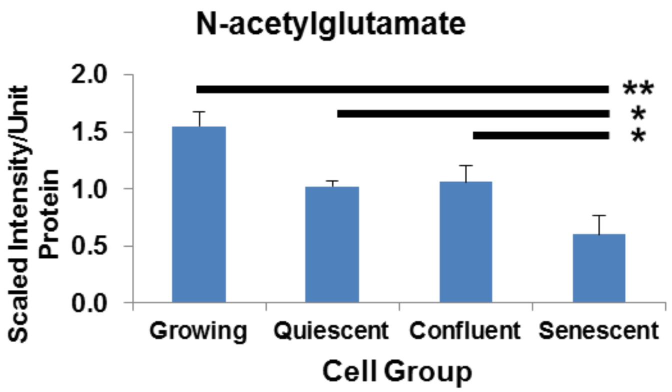
N-acetylglutamate
**
*
*
Scaled Intensity/Unit Protein
2.0
1.5
1.0
0.5
0.0
Growing
Quiescent
Confluent
Senescent
Cell Group

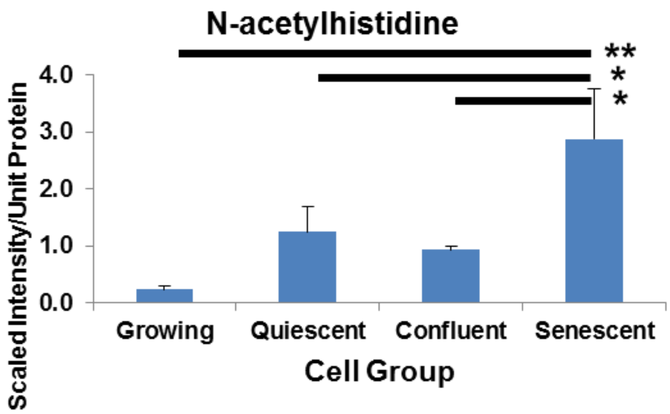
N-acetylhistidine
**
*
*
Scaled Intensity/Unit Protein
4.0
3.0
2.0
1.0
0.0
Growing
Quiescent
Confluent
Senescent
Cell Group

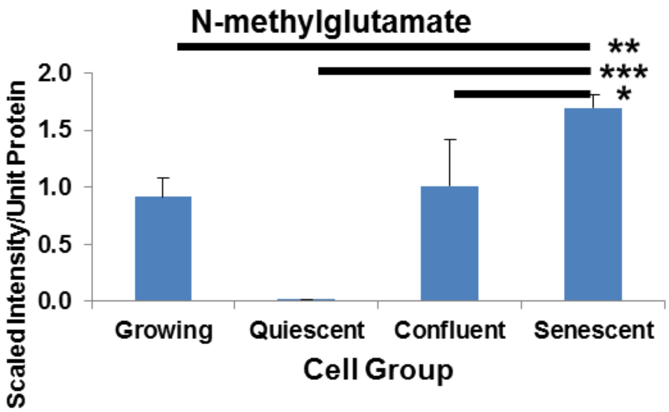
N-methylglutamate
**
***
*
Scaled Intensity/Unit Protein
2.0
1.5
1.0
0.5
0.0
Growing
Quiescent
Confluent
Senescent
Cell Group

Supplementary Fig. S8. Modulation of creatine metabolism and miscellaneous amino acid metabolites in PEsen NHOF-1 cells relative to growing, quiescent and confluent cells.

The Figure shows the miscellaneous amino acid metabolites that are strongly upregulated in senescent cells. In particular creatine and creatine phosphate are strongly and specifically upregulated. Levels of each metabolite normalised to cell protein content +/- standard deviation in growing, quiescent, confluent and PEsen NHOF-1 oral fibroblasts. N = 3 per cell group. The symbols are the same as for Supplementary Fig. S2.

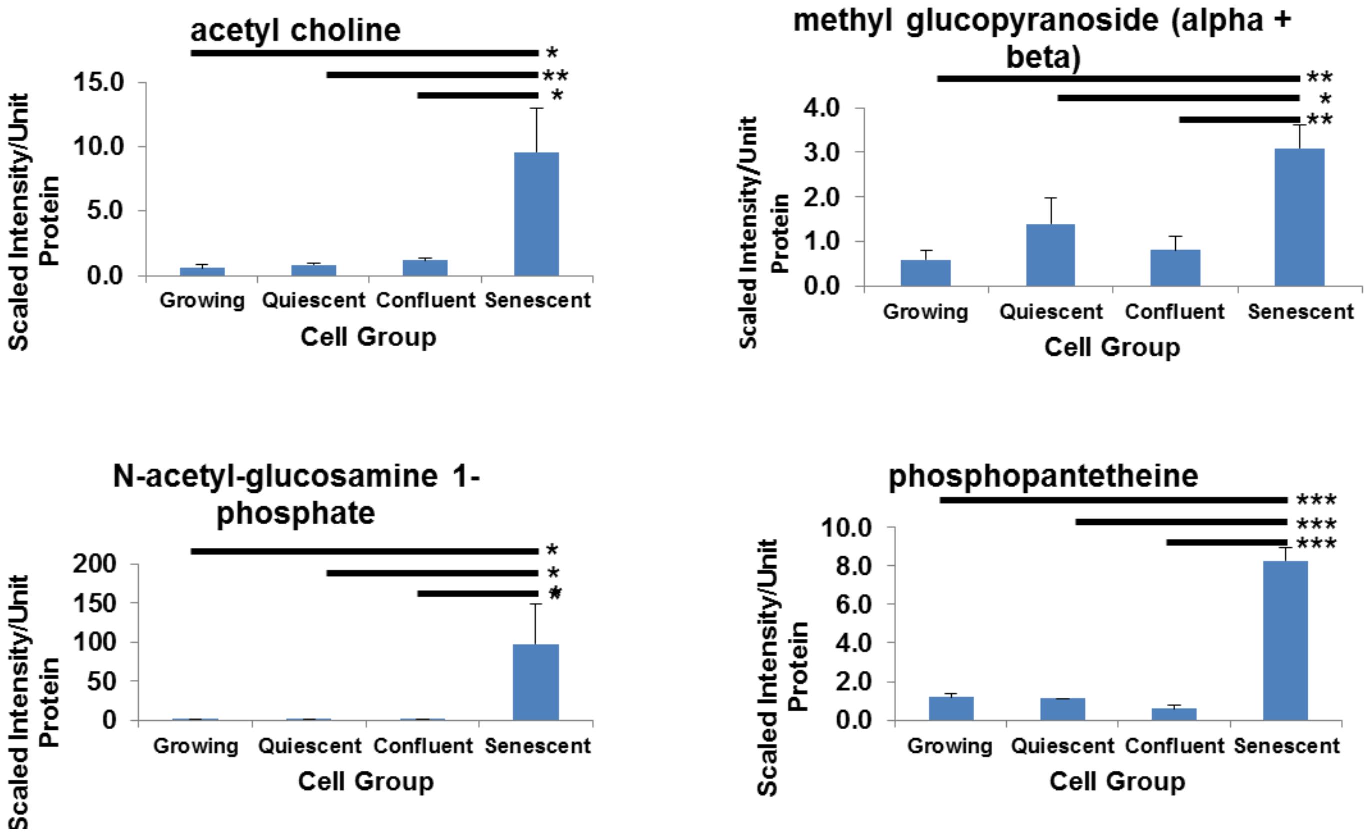

Supplementary Fig. S9. Modulation of miscellaneous metabolites in PEsen NHOF-1 cells relative to growing, quiescent and confluent cells.

The Figure shows the miscellaneous metabolites that are strongly upregulated in senescent cells. Levels of each metabolite normalised to cell protein content +/- standard deviation in growing, quiescent, confluent and PEsen NHOF-1 oral fibroblasts. N = 3 per cell group. The symbols are the same as for Supplementary Fig. S1.

Supplementary References

1. Evans, A. M., et al. Integrated, nontargeted ultrahigh performance liquid chromatography/electrospray ionization tandem mass spectrometry platform for the identification and relative quantification of the small-molecule complement of biological systems. *Anal Chem,* **81** 6656-67 (2009).

2. Sha, W., et al. Metabolomic profiling can predict which humans will develop liver dysfunction when deprived of dietary choline. *FASEB J,* **24** 2962-75 (2010).

3. Dehaven, C. D., Evans, A. M., Dai, H. & Lawton, K. A. Organization of GC/MS and LC/MS metabolomics data into chemical libraries. *J Cheminform,* **2** 9 (2010).

4. James, E. L., et al. Senescent human fibroblasts show increased glycolysis and redox homeostasis with extracellular metabolomes that overlap with those of irreparable DNA damage, aging, and disease. *J Proteome Res,* **14** 1854-71 (2015).

5. Pitiyage, G. N., et al. Senescent mesenchymal cells accumulate in human fibrosis by a telomere-independent mechanism and ameliorate fibrosis through matrix metalloproteinases. *J Pathol,* **223** 604-17 (2011).

6. Nikolakopoulou, Z., Nteliopoulos, G., Michael-Titus, A. T. & Parkinson, E. K. Omega-3 polyunsaturated fatty acids selectively inhibit growth in neoplastic oral keratinocytes by differentially activating ERK1/2. *Carcinogenesis,* **34** 2716-25 (2013).
